# Investigating a Dual-Channel Network in a Sustainable Closed-Loop Supply Chain Considering Energy Sources and Consumption Tax

**DOI:** 10.3390/s22093547

**Published:** 2022-05-06

**Authors:** Mehran Gharye Mirzaei, Fariba Goodarzian, Saeid Maddah, Ajith Abraham, Lubna Abdelkareim Gabralla

**Affiliations:** 1 Department of Industrial Engineering, K. N. Toosi University of Technology, Tehran 1969764499, Iran; m.gharyemirzaei@alumni.kntu.ac.ir; 2 Organization Engineering Group, School of Engineering, University of Seville, Camino de los Descubrimientos s/n, 41092 Seville, Spain; 3 Machine Intelligence Research Labs (MIR Labs), Scientific Network for Innovation and Research Excellence, 11, 3rd Street NW, P.O. Box 2259, Auburn, WA 98071, USA; ajith.abraham@ieee.org; 4 Department of Occupational Health Engineering, Faculty of Health, Thran Medical Sciences, Islamic Azad University, Tehran 1468763785, Iran; maddah.sd@gmail.com; 5 Department of Computer Science and Information Technology, Princess Nourah Bint Abdulrahman University, P.O. Box 84428, Riyadh 11671, Saudi Arabia; lagabralla@pnu.edu.sa

**Keywords:** agricultural products’ supply chain optimization, mathematical modeling, metaheuristic algorithms, multi-objective optimization

## Abstract

This paper proposes a dual-channel network of a sustainable Closed-Loop Supply Chain (CLSC) for rice considering energy sources and consumption tax. A Mixed Integer Linear Programming (MILP) model is formulated for optimizing the total cost, the amount of pollutants, and the number of job opportunities created in the proposed supply chain network under the uncertainty of cost, supply, and demand. In addition, to deal with uncertainty, fuzzy logic is used. Moreover, four multi-objective metaheuristic algorithms are employed to solve the model, which include a novel multi-objective version of the recently proposed metaheuristic algorithm known as Multi-Objective Reptile Search Optimizer (MORSO), Multi-Objective Simulated Annealing (MOSA), Multi-Objective Particle Swarm Optimization (MOPSO), and Multi-Objective Grey Wolf (MOGWO). All the algorithms are evaluated using LP-metric in small sizes and their results and performance are compared based on criteria such as Max Spread (MS), Spread of Non-Dominance Solution (SNS), the number of Pareto solutions (NPS), Mean Ideal Distance (MID), and CPU time. In addition, to achieve better results, the parameters of all algorithms are tuned by the Taguchi method. The programmed model is implemented using a real case study in Iran to confirm its accuracy and efficiency. To further evaluate the current model, some key parameters are subject to sensitivity analysis. Empirical results indicate that MORSO performed very well and by constructing solar panel sites and producing energy out of rice waste up to 19% of electricity can be saved.

## 1. Introduction

In recent years, Agricultural Supply Chain (ASC) management has been the focus of many researchers due to its limited shelf life and the variety of demand and cost [1]. Moreover, the concept of sustainability in the logistics of these products with the goal to consider the environmental, economic, and social dimensions has been simultaneously set forth as another significant issue in the world [2,3]. Sustainable agriculture provides a potential solution to enable agricultural systems to feed a growing population within the changing environmental conditions [4].

**Figure 1:**
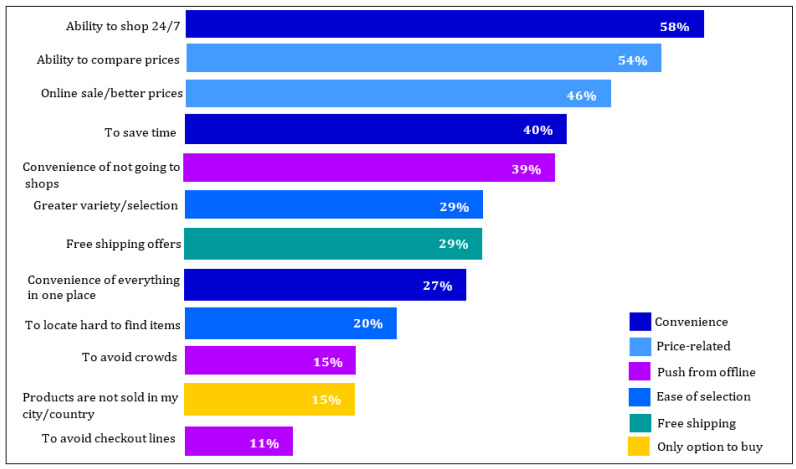
Some online shopping merits (www.smartinsights.com (accessed on 3 March 2022)).

Besides the concept of sustainability, today, electronic commerce (E-commerce) is remarkably influencing supply chain management and involves such benefits as ensuring products are up-to-date, pricing information, boosting communications speed, and so on [5,6]. For this reason, it has attracted lots of customers [7]. Figure 1 displays some of the reasons for consumers to shop online (www.smartinsights.com (accessed on 3 March 2022)).

**Figure 2:**
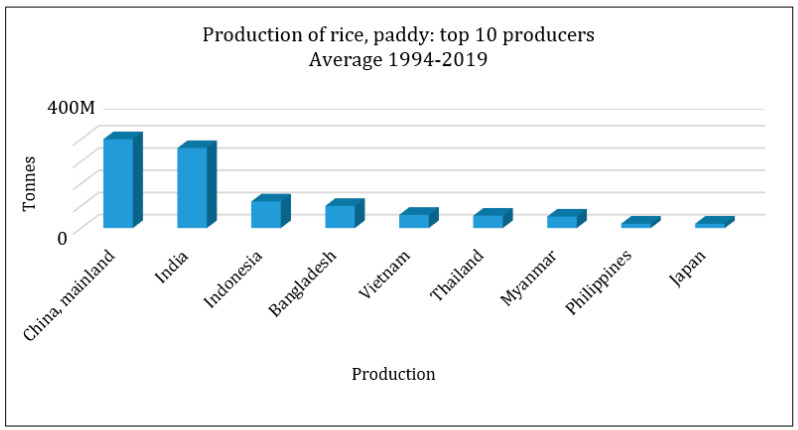
Major rice producing countries (www.fao.org (accessed on 3 March 2022)).

**Figure 3:**
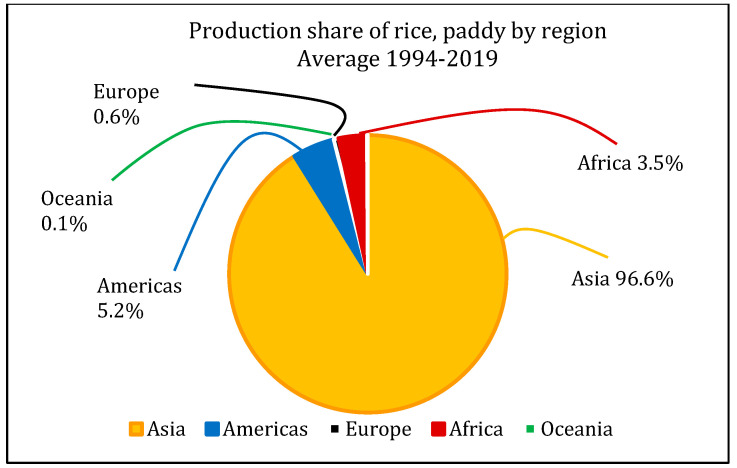
Rice production share in each continent (www.fao.org (accessed on 3 March 2022)).

The agricultural products industry involves the most practical and challenging applications in this field. Among such products, rice is a product consumed as the main part of meals in most parts of the world. Over half of the world population feeds on this basic grain, and this product plays a critical role in boosting the economies of the producing nations [8]. In 2018, global rice production was 782 million tons, 50% of which belonged to China and India, as depicted in Figure 2 and Figure 3 (www.fao.org (accessed on 3 March 2022)).

**Figure 4:**
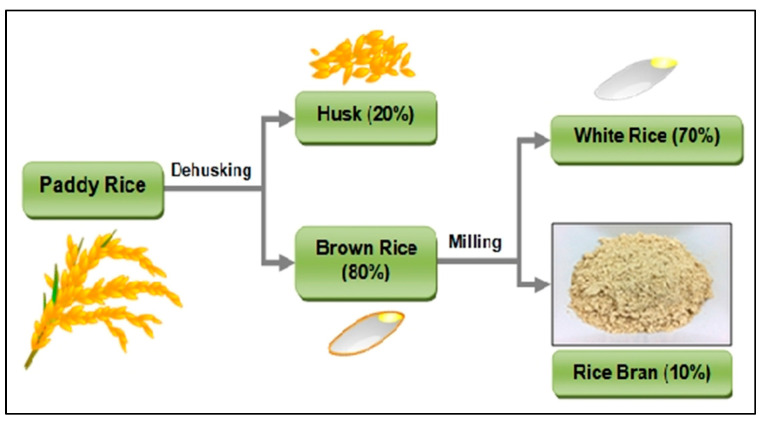
Paddy rice and its components.

**Figure 5:**
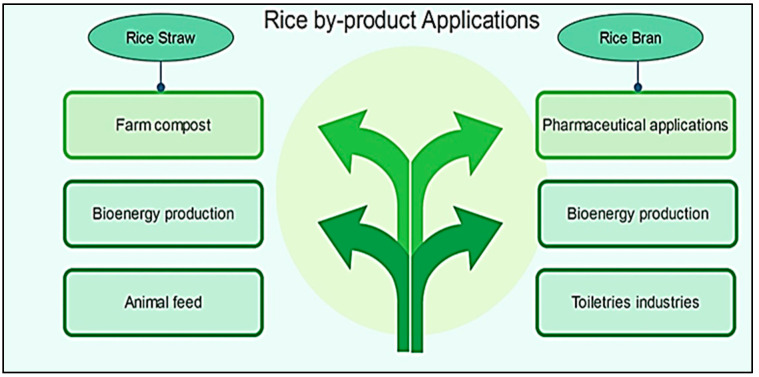
Some rice by-product applications.

In addition to consuming rice as a main meal, the bran is widely utilized in the production of toiletry and pharmaceutical industries. Rice straw can also be used in the production of bio-energy and compost [9] (see Figure 4 and Figure 5).

Most of the rice-producing countries suffer heavy and frequent losses in crop harvest because of poor maintenance of technologies, inefficient supply chains, and farmers’ incapability to market the products. In research conducted by the World Bank and FAO, it has been claimed that, on average, around 8% to 26% of the rice produced in developing nations is destroyed due to poor facilities and post-harvest problems (www.fao.org (accessed on 3 March 2022)).

**Figure 6:**
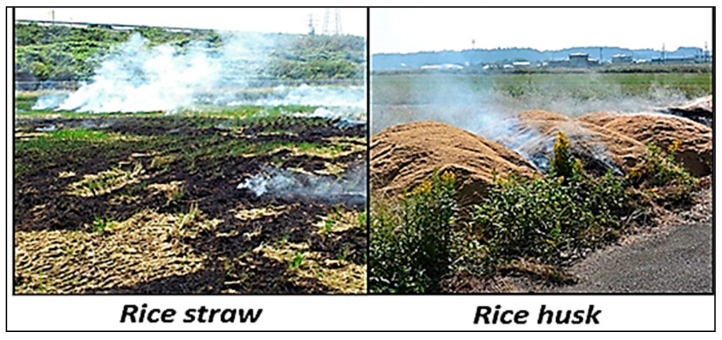
Rice waste disposal and its environmental impacts.

**Figure 7:**
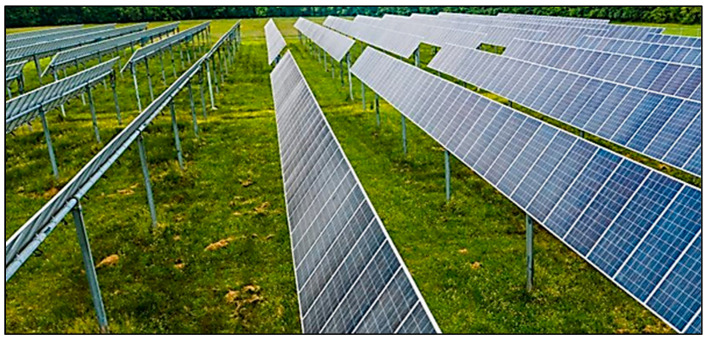
Using solar panels in agriculture sector.

In addition, the rice-processing industry is heavily dependent on energy consumption. With environmental concerns such as greenhouse gas emissions, there is an urgent need to switch to non-carbon, renewable, and clean energy sources such as solar and biomass energy for rice processing [10]. Recent studies revealed that rice waste such as the husk and straw has great potential for electricity generation [8]. Instead of being disposed, rice waste that is properly used can bring about positive environmental effects (see Figure 6 and Figure 7). Selecting an optimal combination out of traditional and renewable energy sources for rice production and processing can slightly lower some of the energy supply problems in this area [11].

Besides supplying energy sources, another important issue that decision-makers and managers may run into when designing a rice supply chain network is the uncertainty of decision making. Uncertainty influences the composition and coordination of the rice supply chain and may take place in several ways, such as the uncertainty in supply and demand. Under real-world conditions, rice supply and customer demand are some of the most common uncertain parameters due to the lack of sufficient information. The high uncertainty level in the supply chain brings about a challenging situation for predicting the future. Fuzzy logic is one of the approaches that is effectively modeling the parameter under uncertainty. The results indicate that fuzzy optimization is a proper option for decision making under uncertainty due to its potential to deal with uncertainty [7].

Pursuant to the mentioned subjects, in this study, optimizing the dual-channel CLSC network of rice is investigated while considering energy sources. Hence, a Mixed Integer Linear Programming (MILP) model is formulated for optimizing the total costs, the quantity of pollutants, and the number of job opportunities created in the proposed logistics network under uncertainty over the cost of purchase and supply and demand. The model aims to locate the proper sites for the construction of solar panels and bio-refineries and to determine the optimal product flow and its waste among the network facilities and the inventory level during each period while considering sustainability factors. Furthermore, fuzzy logic is applied for dealing with uncertainty. After that, four multi-objective metaheuristic algorithms, i.e., MORSO, MOSA, MOPSO, and MOGWO, are used to solve the model, and their results and performance are compared based on several criteria. Finally, the programmed model is implemented on a real case study in Iran in order to verify its accuracy and efficiency. The novel aspects reported in this paper are mainly as follows:

➢ Considering the energy sources are rarely observed in the ASC optimization models, in this paper, a sustainable double-channel CLSC network for rice product considering energy sources and tax on energy consumption is designed. Then, a new MILP model is formulated for optimizing the total costs, the quantity of pollutants, and the job opportunities created in the rice CLSC in a fuzzy environment.
➢ After that, to solve the model and find the Pareto solutions, a new multi-objective version of the recently released algorithm termed Multi-Objective Reptile Search Optimizer (MORSO) is employed to solve the proposed model in high dimensions. Next, its results and performance are compared to MOSA, MOGWO, and MOPSO based on criteria such as MS, SNS, MID, and CPU time.

The present paper is organized into six sections. In the continuation, the literature is reviewed in Section 2. The problem and the mathematical model are stated in Section 3. The solution approach is addressed in Section 4. The proposed model’s accuracy is verified by implementing it onto a real case study in Iran in Section 5. Moreover, the metaheuristic algorithm parameters are tuned and the results of solving the model are analyzed and compared. For further analyzing the model, sensitivity analysis is run on the key parameters. The conclusions and suggestions for research development are presented in Section 6.

## 2. Literature Review

As stated before, producing rice is both economically and socially critical for most rice-producing countries. However, there are few studies related to the logistics optimization of this product. Thus, in this section, the studies performed on supply chain optimization of other agricultural products are reviewed in order to discover the existing research gap. Therefore, the published papers related to this area are reviewed in two sub-sections called ASC optimization and ASC sustainability.

### 2.1. ASC Optimization

As mentioned in the prior sections, agriculture is one of the most salient sectors influencing the economies of many countries. As a result, many researchers have made efforts to enhance the logistics management of agricultural products using novel methods such as operational research. One of the primary studies on programming models in ASC was conducted by [12]. They conducted research on various agricultural products, both perishable and non-perishable, and vegetables. The authors of [13] addressed the production and distribution of perishable products and presented a mathematical model for optimizing the product’s quality and freshness.

The author of [14] presented an integrated model for investment-related decision-making in the fruit and vegetable industry. The authors of [15] proposed a transportation programming model for a fruit supply chain in which a fruit logistics center was established by various storage centers to meet demand during the non-harvest seasons. The authors of [16] proposed a mathematical model for optimizing the cost of purchasing, transporting, and storing fresh agricultural products. They tested the performance of their proposed model on a real case study in an apple juice factory.

The authors of [17] presented a multi-period, non-linear mathematical programming model for optimizing the transportation and operational costs in the logistics system of wheat. The authors of [18] developed a new mathematical model with the goal of reducing the costs of citrus CLSC and maximizing responsiveness to customers’ demand in forward and reverse flow. The work in [19] designed a logistics network for a wheat supply chain and proposed a mathematical programming model for minimizing the total costs under demand uncertainty. They used robust optimization to deal with uncertainty.

The authors of [20] presented a non-linear mathematical model for optimizing the costs and the total profit of an agricultural logistics network. They employed several metaheuristic algorithms such as NSGA-II, MOICA, and MOPSO. In other research, the authors of [21] presented a mathematical model for optimizing the costs in a rice supply chain network. They applied some well-known metaheuristic algorithms for solving the proposed model. Their derived results denoted the proposed model and their solution methods as being valid, practical, and effective.

The authors of [22] developed a novel CLSC network for walnut product. Then, they formulated an MILP model for minimizing the total costs of the proposed network. In addition, they used several metaheuristic algorithms for solving the presented model. Their results indicated their solution approach and the model being valid. Developing a CLSC network for the avocado industry was another study in [23]. An MILP mathematical model was formulated for optimizing the total costs and the number of the job opportunities created in the proposed network. They employed exact methods to solve the presented model. The authors of [24] developed a multi-echelon supply chain network for the sugarcane industry. Following that, they formulated an MILP mathematical model to minimize the total network costs, and due to its NP-hard nature, they applied some hybrid metaheuristic algorithms to solve it. Their results revealed the efficiency of the model and the solution approach.

### 2.2. ASC Sustainability

In recent years, the sustainability concept in supply chains, especially the logistics of agricultural products, has appealed to many researchers. The environmental and social effects of producing and consuming agricultural products have led the decision-makers toward sustainable management, and several related studies have been performed by researchers. For instance, the authors of [25] presented a two-objective mathematical model to minimize demand overruns and to maximize profits in a pear logistics network. They used a lexicographic method to solve their model. The authors of [26] presented a sustainable, multi-objective MILP mathematical model for optimizing the CLSC network of mushroom production by considering a variety of recycling technologies. Their results revealed that using technology increases the overall profit by up to 12% and reduces emissions by about 28%. The authors of [27] presented a mathematical model for a wheat supply chain network, pursuing the goal of optimizing the total costs (including the fixed costs for selecting the suppliers and locating the warehouses and the variable costs for maintenance, transportation, and production). They employed GAMS software for solving the model, the results of which indicated that their proposed model can be used in making decisions about wheat import and distribution.

The authors of [28] proposed a multi-objective MILP model for optimizing wheat production in Spain while considering the environmental impacts.

The authors of [29] analyzed the agro-food supply chain design, focusing on the sustainable dimensions in their multi-objective model. The work in [30] presented a multi-objective non-linear programming model for the sustainable supply chain of perishable agro-food products produced through organic and non-organic methods. Their goal was to strike a balance between the production and consumption of the organic and non-organic products and reduce the costs, lower environmental degradation, and increase the levels of consumer health, wherein they used the Epsilon constraint method to solve the model.

The work in [31] presented a mathematical programming model with the goal of optimizing the costs in an apple supply chain and investigated the environmental effects in their network.

The work in [2] designed an MILP model to optimize the costs, meet the demand, and reduce CO_2_ emissions in a citrus CLSC network. Moreover, they employed three metaheuristic algorithms called MOTGA, NRGA, and NSGA-II. Their results and analysis suggested MOTGA outperformed in this regard.

The authors of [32] prepared a multi-objective mathematical programming model for optimizing the costs, water consumption, and the number of job opportunities created in a wheat logistics network under demand uncertainty. Next, they used a simulation approach for estimating the demand. The authors of [33] presented a bi-objective mathematical model for optimizing the profit and the quantity of the pollutants in a green pistachio supply chain. In their model, the demand and costs were assumed as uncertain. They used a robust possibilistic programming model to deal with uncertainty. In addition, they employed the Epsilon constraint method to solve the model. The work in [27] proposed a multi-objective MILP model to optimize the costs and the quantity of the pollutants emitted in a date supply chain. They used the LP-metric method and some metaheuristic algorithms to solve the model.

### 2.3. Research Gap and Motivation for Research

In the current study, in order to come across the study gap, we analyzed several papers about ASC optimization. A brief review of some related studies is given in Table 1. Briefly, by investigating the mentioned papers in the table above, it can be concluded that few papers have been published about rice logistics optimization. Furthermore, the sustainability dimensions in ASC optimization have rarely been considered concurrently. The agricultural sector, on the one hand, has enormous environmental effects and plays a critical role in climatic changes, water scarcity, land degradation, deforestation, and other processes, and on the other hand, the income of many rural people depends on it and the sector provides several job opportunities for them. Thus, considering the economic, environmental, and social factors simultaneously and creating a trade-off among them greatly help to improve the ASC network, particularly for rice, an issue that has rarely been analyzed. Besides the sustainability concept, uncertainty has seldom been considered in the optimization models in the agricultural sector. Undoubtedly, the supply chain of agricultural products is challenging, with various uncertainties in the real world, and therefore it is necessary to employ proper approaches for modeling under uncertain conditions for successful optimization and effective decision making in the supply chain.

Furthermore, considering the energy sources is rarely observed in ASC optimization models. There is no doubt that the processing of agricultural products, especially rice, in various processes, including its own processing, requires electrical energy. Processing stages are significantly energy-consuming, which exerts environmental effects. Thus, it urgently necessitates employing renewable and clean energy sources for rice processing. As a result, selecting an optimal combination of traditional and renewable energy used for processing can boost energy efficiency and mitigate the environmental consequences. To the best of our knowledge, the present study is one of the first papers that introduces a mathematical model with the goal of optimally using rice waste and properly locating solar panel sites and bio-refineries for minimizing the fixed and operational costs intending to generate electricity.

Moreover, when investigating the relevant studies, the traditional and online channels in the ASC network design have rarely been analyzed. Today, with the expansion of E-commerce, most consumers are increasingly becoming fond of purchasing agricultural products online. The online purchase of agricultural products has attracted many researchers due to advantages such as more competitive prices, timely purchase, easy product delivery, and fast product delivery [36].

In order to bridge the research gap in this study, we introduce a dual-channel CLSC network, including the traditional and online purchasing of rice, while taking into account the energy sources. Then, we present a three-objective mathematical model for optimizing the costs, the quantity of the emitted pollutants, and the number of job opportunities created in the rice supply chain network in a fuzzy environment. Next, the designed model is solved via some multi-objective metaheuristic algorithms and their results and performance are compared. Furthermore, using a case study in Iran, the accuracy of the programmed model is surveyed. In the following sections, the problem model and its solution method are presented and described.

## 3. Problem Statement

**Figure 8:**
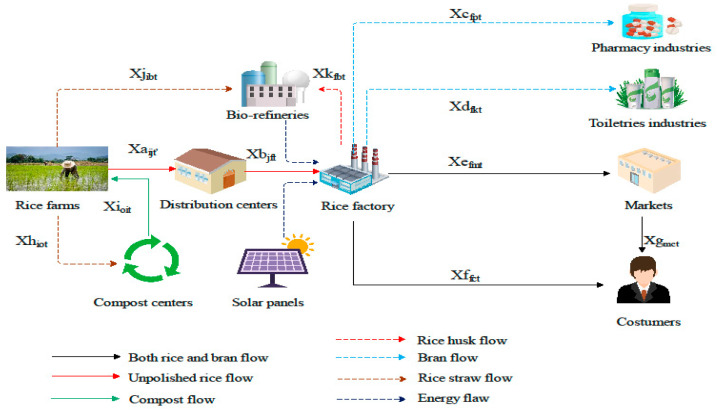
Proposed rice logistics network.

**Figure 9:**
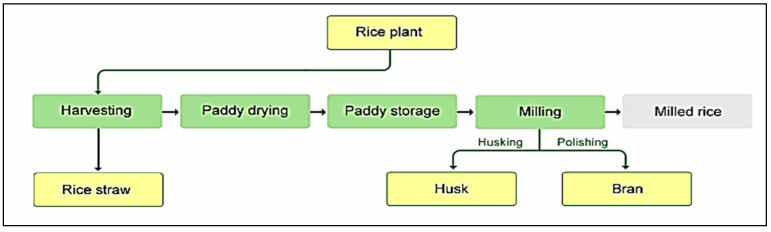
Rice production stages.

In this section, the logistics network and the proposed mathematical model for optimizing the objectives are described. The proposed multi-period network in Figure 8 involves the producers (farmers), the distribution centers, the solar panel sites, the bio-refineries, the rice factories, the warehouses, the markets (retailers), the customers, and the toiletry and pharmaceutical industries. In this network, the farmers harvest rice in two periods and transfer the unprocessed rice (paddy) from farms to the distribution centers. Moreover, rice harvest-produced waste, i.e., the straw, is transferred to the recycling centers and bio-refineries to produce compost and convert it into electricity, respectively. The paddy from the farmers is transferred to the distribution centers in two months because the harvest is completed in two months at a maximum level. Furthermore, the maximum shelf life of rice in the distribution centers is 6 months and, from there, it is transferred to the rice factories for processing. Figure 9 depicts the production and processing stages of rice. As rice processing requires a lot of electricity, some points have been considered as potential sites for constructing solar panels and bio-refineries with the goal of supplying energy.

Moreover, rice processing-induced waste, i.e., rice husk, is transferred to the bio-refineries due to its potential to generate electricity. Ultimately, both the processed rice and the bran are dispatched from the factory to both the markets and directly to the consumers to satisfy their demand. As observed in Figure 8, a dual channel was introduced in the proposed supply chain network in which the customers can purchase rice and bran both online and directly from the warehouse and from the market in a traditional manner. Some rice bran can also be sent to the toiletry and pharmaceutical industries. It is worth noting that the location of all facilities except for the new distribution centers, the new recycling centers, the solar panel sites, and the bio-refineries, is fixed. The goal behind this model is to appropriately locate the construction of solar panels, the bio-refineries, and the recycling centers to create a balance among the total cost, the quantity of the pollutants, and the number of the job opportunities created by considering the energy sources in the network so that the consumers’ demand is met. The indicators, the parameters, and the variables of decision making are presented in the Appendix A, and here, the mathematical model is explained. The proposed model’s assumptions are the following:

- Transportation costs between the network facilities are consistent with the distance.
- Processing centers have limited storage capacity.
- Deficiency cost has not been considered.
- Rice production capacity in factories is also limited.
- Excess electrical energy should be connected to the mains (grid) electricity.

### 3.1. Problem’s Model

After describing the offered problem and assumptions, the expanded multi-objective mathematical model is formulated as the following:

(1a) $$Min\;\;z_{1}=\sum_{r\in R}\sum_{c\in C}\sum_{t\in T}\sum_{p\in P}\tilde{prm_{pmt}}\times Xg_{prct}+\sum_{f\in F}\sum_{c\in C}\sum_{t\in T}\sum_{p\in P}\tilde{prf_{pft}}\times Xf_{pfct}$$

(1b) $$+\underset{j\in J_{2}}{\sum }fcj_{j_{\;}}\times w_{j}+\underset{s\in S}{\sum }fcs_{s}\times v_{s}+\underset{b\in B}{\sum }fcb_{b}\times z_{b}+\underset{o\in O2}{\sum }fco_{o}\times Q_{o}$$

(1c) $$\begin{array}{l} +CTV\times (\sum_{i\in I}\sum_{j\in J}\sum_{t\in T}disa_{ij}\times Xa_{ijt}+\sum_{j\in J}\sum_{f\in F}\sum_{t\in T}disb_{jf}\times Xb_{jft}+\\ \sum_{f\in F}\sum_{v\in V}\sum_{t\in T}disc_{fv}\times Xc_{fvt}+\sum_{f\in F}\sum_{k\in K}\sum_{t\in T}disd_{fk}\times Xd_{fkt}+\sum_{p\in P}\sum_{f\in F}\sum_{m\in M}\sum_{t\in T}dise_{fm}\times Xe_{pfmt}\\ +\sum_{p\in P}\sum_{f\in F}\sum_{c\in C}\sum_{t\in T}disf_{fc}\times Xf_{pfct}+\sum_{p\in P}\sum_{m\in M}\sum_{c\in C}\sum_{t\in T}disg_{mc}\times Xg_{pmct}+\sum_{o\in O}\sum_{i\in I}\sum_{t\in T}dish_{oi}\times Xh_{oit}\\ +\sum_{i\in I}\sum_{o\in O}\sum_{t\in T}disi_{io}\times Xi_{iot}+\sum_{b\in B}\sum_{i\in I}\sum_{t\in T}disj_{ib}\times Xj_{ibt}+\sum_{f\in F}\sum_{b\in B}\sum_{t\in T}disk_{fb}\times Xk_{fbt}) \end{array}$$

(1d) $$+\sum_{i\in I}\sum_{t^{\prime }\in T}cpa_{i}\times Xq_{it^{\prime }}+\sum_{j\in J}\sum_{t^{\prime }\in T}cpa_{jt}\times Xhj_{jt}+\sum_{i\in I}\sum_{o\in O}\sum_{t\in T}cpc_{o}\times Xh_{oit^{\prime }}$$

(1e) $$+\underset{r\in R}{\sum }\underset{t\in T}{\sum }\;\tilde{cta}x_{r}\times XSe_{rt}+\underset{f\in F}{\sum }\underset{p\in P}{\sum }\underset{r\in R}{\sum }\underset{t\in T}{\sum }\tilde{cp}t_{pfr}\times Xrh_{fprt}$$

(2a) $$Min\;\;z_{2}=\underset{b\in B}{\sum }\pi b_{b}\times Z_{b}+\underset{u\in U}{\sum }\pi u_{u}\times V_{u}+\underset{o\in O2}{\sum }\pi o_{o}\times W_{o}+\underset{f\in F}{\sum }\underset{p\in P}{\sum }\underset{r\in R}{\sum }\underset{t\in T}{\sum }\pi p_{p}\times Xrh_{fprt}$$

(2b) $$\begin{array}{l} +\sum_{i\in I}\sum_{j\in J}\sum_{t^{\prime }\in T}\varphi \times RF\times (\sum_{i\in I}\sum_{j\in J}\sum_{t^{\prime }\in T}disa_{ij}\times \frac{Xa_{ijt^{\prime }}}{capv}+\sum_{j\in J}\sum_{f\in F}\sum_{t\in T}disb_{jf}\times \frac{Xb_{jft}}{capv}\\ +\sum_{f\in F}\sum_{v\in V}\sum_{t\in T}disc_{fv}\times \frac{Xc_{fvt}}{capv}+\sum_{f\in F}\sum_{b\in B}\sum_{t\in T}disd_{fk}\times \frac{Xd_{fkt}}{capv}+\sum_{p\in P}\sum_{f\in F}\sum_{m\in M}\sum_{t\in T}dise_{fm}\times \frac{Xe_{pfmt}}{capv}\\ +\sum_{p\in P}\sum_{f\in F}\sum_{c\in C}\sum_{t\in T}disf_{fc}\times \frac{Xf_{pfct}}{capv}+\sum_{p\in P}\sum_{m\in M}\sum_{c\in C}\sum_{t\in T}disg_{mc}\times \frac{Xg_{pmct}}{capv}+\sum_{i\in I}\sum_{o\in O}\sum_{t\in T}dish_{io}\times \frac{Xh_{oit}}{capv}\\ +\sum_{o\in O}\sum_{i\in I}\sum_{t\in T}disi_{oi}\times \frac{Xi_{iot}}{capv}+\sum_{o\in O}\sum_{b\in B}\sum_{t\in T}disj_{ib}\times \frac{Xj_{ibt}}{capv}+\sum_{f\in F}\sum_{b\in B}\sum_{t\in T}disk_{fb}\times \frac{Xk_{fbt}}{capv} \end{array}$$

(3) $$Max\;\;z_{3}=\sum_{j\in J2}FJ_{j}\times W_{j}+\sum_{i\in I}\sum_{t^{\prime }\in T}VJI_{i}\times \frac{Xq_{it^{\prime }}}{cap_{it^{\prime }}}+\sum_{r\in R}\sum_{f\in F}\sum_{t\in T}\sum_{p\in P}VJQ_{pf}\times \frac{Xrh_{fprt}}{pcapf_{pft}}$$

The model’s objective function z_1_ related to the economic aspect of sustainabilty maximizes the total cost. This amount of the sum of the cost refers to the consumers’ rice purchase (1a), the cost of establishing new facilities (1b), transportation cost (1c), the cost of production for farmers, the cost of maintaining (storing) the product, and the cost of production for the rice factories (1d), and the cost of energy tax (1e). Objective function z_2_ related to the environmental aspect of sustainability minimizes the total amount of CO_2_ emitted from constructing the new facilities (2a) and the vehicles traveling among the network facilities (2b). The vehicle has a certain capacity (capv) that is based on the quantity of the product transported in each stage (for instance, n for the first time $(n=Xa_{ijt^{\prime }}/\;capv_{\;}$)), the number of transportation times is determined; this vehicle needs a certain amount of fuel per k(φ) for transportion, and when this fuel is used by the vehicle, a certain amount of CO_2_ per km (*RF*) is emitted.

Objective function z_3_ is associated with the third aspect of sustainability and maximizes the fixed and variable job opportunities. The fixed job opportunities are independent of production quantity, but the variable job opportunities are dependent on the production quantity. This objective function consists of three segments: the 1st one optimizes the fixed job opportunities created by opening the new facilities; the 2nd one optimizes the variable job opportunities of rice production in the farms; the 3rd one optimizes the variable job opportunities of rice production in the factories. Our target behind presenting this model was to strike a balance among the costs, the quantity of the pollutants, and the number of the fixed and variable job opportunities. This model’s constraints are the following:

**Constraints:**

(4) $$\begin{array}{ll} Xq_{it^{\prime }}\leq \tilde{cap_{it^{\prime }}}\;\;\;\;\;\;\;\;\;\;\;\;\;\;\;\;\;\;\;\;\;\;\;\;\;\;\;\;\;\;\;\;\;\;\;\;\;\;\;\;\;\;\;\;\;\;\;\;\;\;\;\;\;\;\;\;\;\;\;\;\;\;\;\;\;\;\;\; & \forall i\in I,t^{\prime }\;\in T\;\;\; \end{array}$$

(5) $$\begin{array}{ll} \sum_{i\in I}\sum_{t^{\prime }\in T^{\prime }}Xa_{ijt^{\prime }}\geq \sum_{k\in K}\sum_{t\in T}Xb_{jft}\;\;\;\;\;\;\;\;\;\;\;\;\;\;\;\;\;\;\;\;\;\;\;\;\;\;\;\;\;\;\; & \forall j\in J\;\;\;t,t^{\prime }\in T \end{array}$$

(6) $$Xhj_{jt}=Xhj_{j,t-1}+\underset{i\in I}{\sum }Xa_{ijt^{\prime }}\;-\underset{k\in K}{\sum }Xb_{jft}\;\;\;\;\;\;\;\;\;\;\;\;\;\;\;\;\;\;\;\;\forall j\in J\;\;t,\;t^{\prime }\in \;T^{\prime }$$

(7) $$\underset{i\in I}{\sum }Xa_{ijt^{\prime }}\leq M\times W_{j_{\;}}\;\;\;\;\;\;\;\;\;\;\;\;\;\;\;\;\;\;\;\;\;\;\;\;\;\;\;\;\;\;\;\;\;\;\;\;\;\;\;\;\;\;\;\;\;\;\;\;\;\;\;\;\;\;\;\;\;\;\;\forall t\in T,j\in J_{2}$$

(8) $$\underset{j\in J}{\sum }{Xb}_{\mathrm{jft}}\leq {\mathrm{capf}}_{f}\;\;\;\;\;\;\;\;\;\;\;\;\;\;\;\;\;\;\;\;\;\;\;\;\;\;\;\;\;\;\;\;\;\;\;\;\;\;\;\;\;\;\;\;\;\;\;\;\;\;\;\;\;\;\;\;\;\;\;\forall f\in F,t\in T$$

(9) $$\;\;\;\underset{f\in F}{\sum }{Xf}_{\mathrm{pfct}}\geq {\tilde{\mathrm{dco}}}_{\mathrm{pct}}\;\;\;\;\;\;\;\;\;\;\;\;\;\;\;\;\;\;\;\;\;\;\;\;\;\;\;\;\;\;\;\;\;\;\;\;\;\;\;\;\;\;\;\;\;\;\;\;\;\;\;\;\;\;\;\;\;\;\;\;\forall c\in C,t\;\in T,p\in P\;$$

(10) $$\underset{k\in K}{\sum }{\mathrm{Xg}}_{\mathrm{prct}}\geq {\tilde{\mathrm{dct}}}_{\mathrm{pct}}\;\;\;\;\;\;\;\;\;\;\;\;\;\;\;\;\;\;\;\;\;\;\;\;\;\;\;\;\;\;\;\;\;\;\;\;\;\;\;\;\;\;\;\;\;\;\;\;\;\;\;\;\;\;\;\;\;\;\;\;\forall c\in C,t\;\in T$$

(11) $$\underset{f\in F}{\sum }{\mathrm{Xd}}_{\mathrm{fkt}}\geq {\mathrm{dk}}_{\mathrm{kt}}\;\;\;\;\;\;\;\;\;\;\;\;\;\;\;\;\;\;\;\;\;\;\;\;\;\;\;\;\;\;\;\;\;\;\;\;\;\;\;\;\;\;\;\;\;\;\;\;\;\;\;\;\;\;\;\;\;\;\;\;\forall k\in K,t\;\in T$$

(12) $$\underset{f\in F}{\sum }{\mathrm{Xe}}_{\mathrm{fvt}}\geq {\mathrm{dv}}_{\mathrm{vt}}\;\;\;\;\;\;\;\;\;\;\;\;\;\;\;\;\;\;\;\;\;\;\;\;\;\;\;\;\;\;\;\;\;\;\;\;\;\;\;\;\;\;\;\;\;\;\;\;\;\;\;\;\;\;\;\;\;\;\;\;\;\;\forall u\in U,t\;\in T$$

(13) $$\;\;\;\underset{r\in R}{\sum }{\mathrm{Xrh}}_{\mathrm{fprt}}\leq {\mathrm{pcapf}}_{\mathrm{pft}}\;\;\;\;\;\;\;\;\;\;\;\;\;\;\;\;\;\;\;\;\;\;\;\;\;\;\;\;\;\;\;\;\;\;\;\;\;\;\;\;\;\;\;\;\;\;\;\;\;\;\;\;\;\;\;\;\;\;\forall p\in P,t\;\in T,f\in F\;$$

(14) $$\;\;\;\sum_{j\in J}\sum_{t\in T}Xb_{jft}\times \theta_{p}=\sum_{r\in R}\sum_{t\in T}Xrh_{fprt}\;\;\;\;\;\;\;\;\;\;\;\;\;\;\;\;\;\;\;\;\;\;\;\;\;\;\;\;\;\;\;\;\;\;\;\;\;\forall f\in F,p\in P$$

(15) $$\begin{array}{ll} \;\;\;\;\;\underset{t\in T}{\sum }\underset{r\in R}{\sum }Xrh_{yrft}= & \underset{t\in T}{\sum }\underset{v\in V}{\sum }Xc_{fvt}+\underset{t\in T}{\sum }\underset{k\in K}{\sum }Xd_{fkt}+\underset{t\in T}{\sum }\underset{m\in M}{\sum }Xe_{\begin{array}{c} \begin{array}{c} yfmt \end{array} \end{array}}\\ & +\underset{t\in T}{\sum }\underset{c\in C}{\sum }Xf_{yfct}\;\;\;\;\;\;\;\;\;\;\;\;\;\;\;\;\;\;\;\;\;\;\;\;\;\;\;\;\;\;\;\;\;\;\;\;\;\forall f\in F \end{array}$$

(16) $$\;\;\;\underset{t\in T}{\sum }\underset{r\in R}{\sum }Xrh_{xrft}=\underset{t\in T}{\sum }\underset{m\in M}{\sum }Xe_{xfmt}+\underset{t\in T}{\sum }\underset{c\in C}{\sum }Xf_{xfct}\;\;\;\;\;\;\forall f\in F$$

(17) $$\underset{i\in I}{\sum }\underset{t^{\prime }\in T^{\prime }}{\sum }{\mathrm{Xa}}_{{\mathrm{ijt}}^{\prime }}\leq {\mathrm{capj}}_{j}\;\;\;\;\;\;\;\;\;\;\;\;\;\;\;\;\;\;\;\;\;\;\;\;\;\;\;\;\;\;\;\;\;\;\;\;\;\;\;\;\;\;\;\;\;\;\;\;\;\;\;\;\;\;\forall j\in J\;,\forall t\in T$$

(18) $$Xhj_{jt}\leq {\mathrm{capj}}_{j}\;\;\;\;\;\;\;\;\;\;\;\;\;\;\;\;\;\;\;\;\;\;\;\;\;\;\;\;\;\;\;\;\;\;\;\;\;\;\;\;\;\;\;\;\;\;\;\;\;\;\;\;\;\;\;\;\;\;\;\;\;\;\;\;\;\forall t\in T\;,\;\forall j\in J$$

(19) $$\sum_{j\in J}Xa_{ijt^{\prime }}=\beta \times Xq_{it^{\prime }}\;\;\;\;\;\;\;\;\;\;\;\;\;\;\;\;\;\;\;\;\;\;\;\;\;\;\;\;\;\;\;\;\;\;\;\;\;\;\;\;\;\;\;\;\;\;\;\;\;\;\;\;\;\;\;\;\forall i\in I,\forall t^{\prime }\in T^{\prime }$$

(20) $$\sum_{t\in T}XSe_{bt}\leq M\times Z_{b}\;\;\;\;\;\;\;\;\;\;\;\;\;\;\;\;\;\;\;\;\;\;\;\;\;\;\;\;\;\;\;\;\;\;\;\;\;\;\;\;\;\;\;\;\;\;\;\;\;\;\;\;\;\forall b\in B$$

(21) $$\sum_{t\in T}XSe_{st}\leq M\times V_{s}\;\;\;\;\;\;\;\;\;\;\;\;\;\;\;\;\;\;\;\;\;\;\;\;\;\;\;\;\;\;\;\;\;\;\;\;\;\;\;\;\;\;\;\;\;\;\;\;\;\;\;\;\;\forall s\in S$$

(22) $$\begin{array}{l} \sum_{i\in I}\sum_{t^{\prime }\in T}{\mathrm{Xi}}_{{\mathrm{iot}}^{\prime }}\leq M\times Q_{o}\;\;\;\;\;\;\;\;\;\;\;\;\;\;\;\;\;\;\;\;\;\;\;\;\;\;\;\;\;\;\;\;\;\;\;\;\;\;\;\;\;\;\;\;\;\;\;\;\; \end{array}\forall o\in O_{2}$$

(23) $$\sum_{i\in I}\sum_{t^{\prime }\in T}Xi_{ibt^{\prime }}+\sum_{f\in F}\sum_{t^{\prime }\in T}Xk_{fbt}\leq M\times Z_{b}\;\;\;\;\;\;\;\;\;\;\;\;\;\;\;\;\;\;\;\;\;\;\;\;\;\;\;\forall i\in I,\forall t^{\prime }\in T$$

(24) $$\underset{i\in I}{\sum }\underset{t^{\prime }\in T}{\sum }{\mathrm{Xi}}_{{\mathrm{iot}}^{\prime }}\leq Capo_{o}\;\;\;\;\;\;\;\;\;\;\;\;\;\;\;\;\;\;\;\;\;\;\;\;\;\;\;\;\;\;\;\;\;\;\;\;\;\;\;\;\;\;\;\;\;\;\;\;\;\;\;\;\forall o\in O_{2}\;\;$$

(25) $$\underset{o\in O}{\sum }Xi_{oit}\geq di_{it}\;\forall i\in I\;,\;\;\;\;\;\;\;\;\;\;\;\;\;\;\;\;\;\;\;\;\;\;\;\;\;\;\;\;\;\;\;\;\;\;\;\;\;\;\;\;\;\;\;\;\;\;\;\;\;\;\forall t^{\prime }\in T$$

(26) $$\underset{o\in O}{\sum }{\mathrm{Xh}}_{{\mathrm{iot}}^{\prime }}+\underset{j\in J}{\sum }{\mathrm{Xj}}_{{\mathrm{ibt}}^{\prime }}=\left(1-\beta \right)\times Xq_{it^{\prime }}\;\;\;\;\;\;\;\;\;\;\;\;\;\;\;\;\;\;\;\;\;\;\;\;\forall i\in I\;,\forall t^{\prime }\in T^{\prime }$$

(27) $$\psi \times \sum_{i\in I}\sum_{t\in T}Xh_{iot^{\prime }}=\sum_{i\in I}\sum_{t\in T}Xi_{oit^{\prime }}\;\;\;\;\;\;\;\;\;\;\;\;\;\;\;\;\;\;\;\;\;\;\;\;\;\;\;\;\;\;\;\;\;\;\;\;\forall o\in O$$

(28) $$\lambda \times \underset{i\in I}{\sum }Xj_{ibt}+\delta \times \underset{f\in F}{\sum }Xk_{fbt}=XSe_{bt}\;\;\;\;\;\;\;\;\;\;\;\;\;\;\;\;\;\;\;\;\;\;\forall b\in B\;,\forall t\in T$$

(29) $$\underset{j\in J}{\sum }\underset{t\in T}{\sum }Xb_{jft}\times \eta \;=\underset{b\in B}{\sum }Xk_{fbt}\;\;\;\;\;\;\;\;\;\;\;\;\;\;\;\;\;\;\;\;\;\;\;\;\;\;\;\;\;\;\;\;\;\;\forall t\in T,f\in F$$

(30) $$\sum_{p\in P}\sum_{f\in F}Xrh_{fprt}\times EP=XSe_{rt}\;\;\;\;\;\;\;\;\;\;\;\;\;\;\;\;\;\;\;\;\;\;\;\;\;\;\;\;\;\;\;\;\;\;\;\;\;\;\;\;\forall r\in R,\forall t\in T$$

(31) $$\begin{array}{l} XSe_{rt}\leq capr_{rt} \end{array}\;\;\;\;\;\;\;\;\;\;\;\;\;\;\;\;\;\;\;\;\;\;\;\;\;\;\;\;\;\;\;\;\;\;\;\;\;\;\;\;\;\;\;\;\;\;\;\;\;\;\;\;\;\;\;\;\forall r\in R,\forall t\in T$$

(32) $$\sum_{f\in F}\sum_{t\in T}Xe_{pfmt}=\sum_{c\in T}\sum_{t\in T}Xg_{pmct}\;\;\;\;\;\;\;\;\;\;\;\;\;\;\;\;\;\;\;\;\;\;\;\;\;\;\;\;\;\;\;\;\;\;\;\;\;\;\;\;\;\;\forall m\in M,\forall p\in P$$

(33) $$\begin{array}{l} Xa_{i}{}_{jt^{\prime }},Xb_{jft},Xc_{fvt},Xd_{fkt},Xe_{pfmt},Xf_{pfct},Xg_{pmct},Xh_{iot},Xi_{oit},\\ Xj_{ibt},Xk_{fbt},Xhj_{jt},Xrh_{fprt}Xse_{rt}\geq 0\;\;\;\;\;\;\;\;\;\;\;\;\;W_{j},V_{s},Z_{b},Q_{o}\in \{0,1\} \end{array}$$

Constraint (4) states that the amount of rice plant harvested by the producers is the maximum of their production capacity. Constraint (5) displays the equilibrium in the distribution centers and states that the sum of the input product to each distribution center is larger than or equal to the output product. Constraint (6) balances the inventory of the unprocessed rice in the distribution centers. Constraint (7) states that the condition for dispatching the product to the distribution centers is the establishment of that center. Constraint (8) denotes that the quantity of the unprocessed rice transferred to each factory has to be less than its storage capacity. Constraints (9) and (10) show that the demand for the rice required by the customers has to be met through traditional and online shopping. Constraints (11) and (12) state that the rice bran demanded by the pharmaceutical and toiletry industries has to be satisfied. Constraint (13) indicates that the quantity of the produced product has to be less than the production capacity of each factory. Constraint (14) demonstrates that the amount of rice or bran produced in factories is gained as a fraction of paddy.

Constraints (15) and (16) state that the total bran and rice produced at each factory has to be delivered to the consumers. Constraints (17) and (18) display the storage capacity of the collection centers. Constraint (19) shows that the quantity of the unprocessed rice transported to the distribution centers has to be equal to the amount of the produced rice plant multiplied by its conversion rate to paddy. Constraints (20) and (21) demonstrate that the energy required for processing rice is supplied from a renewable energy source if it is opened. Constraint (22) displays that rice straw is transported to the recycling centers if that center is opened. Constraint (23) states that rice straw and husk are dispatched to the bio-refineries if the center is opened. Constraint (24) shows that the quantity of rice transported to each center has to be less than its storage capacity. Constraint (25) displays the compost demanded by the farmers that has to be met during each period. Constraint (26) demonstrates that the amount of rice straw transferred to the bio-refineries and the recycling centers is a fraction of the quantity of the produced rice plant.

Constraint (27) states that the amount of compost produced in recycling centers is equal to the amount of the received rice straw multiplied by its conversion rate to compost. Constraint (28) demonstrates that the energy generated from rice waste in each bio-refinery is equal to the amount of the received rice straw and husk multiplied by their conversion rate into energy. Constraint (29) indicates that the amount of rice husk transported to the bio-refinery is limited and equal to the amount of the received paddy multiplied by its conversion rate to husk.

Constraint (30) indicates that the quantity of the rice and the produced bran in each factory is bound to the available energy. Constraint (31) shows that the energy generated from each source is bound to the energy generation capacity, and Constraint (32) balances the flow in each market. Finally, Constraint (33) depicts the model’s type of decision variables.

### 3.2. Uncertainty Model

In the proposed model, the parameters of purchase cost, supply, and demand are uncertain. In this section, using the fuzy logic proposed by Jimenez’s method [37], we introduce the uncertain counterpart of the proposed model. As Jimenez’s method does not impose additional inequality constraints, it is efficient in solving fuzzy decision problems [7]. Pursuant to this method, the fuzzy parameters presented in Equations (34) and (35) can be represented by a set of triangular fuzzy numbers for the optimistic, realistic, and pessimistic cases. Regarding Jimenez’s method, the Expected Interval (EI) and the Expected Value (EV) of a fuzzy parameter can be estimated as given in the following:

(34) $$\begin{array}{l} EI\left(\tilde{c}\right)=[E_{1},E_{2}]=[\overset{1}{\underset{0}{\int }}f_{c}{}^{-1}\left(x\right)dx,\;\;\overset{1}{\underset{0}{\int }}g_{c}{}^{-1}\left(x\right)dx]=\\ \overset{1}{\underset{0}{\int }}(x\left(c^{q}-c^{p}\right)+c^{p})dx=\left[\overset{1}{\underset{0}{\int }}(x\left(c^{s}-c^{q}\right)+c^{s}\right)dx]=[\frac{1}{2}\left(c^{w}+c^{q}\right),\frac{1}{2}\left(c^{q}+c^{v}\right)]\\ EV\left(\tilde{c}\right)=\frac{\left(E_{1}{}^{C}+E_{2}{}^{C}\right)}{2}=\frac{c^{w}+2c^{q}+c^{v})}{4} \end{array}$$

The fuzzification of a parameter based on Jimenez’s method can be presented as follows. Suppose a fuzzy parameter of demand in the optimistic, realistic, and pessimistic state including the values $c^{w}=$3, 4, and $c^{v}=$6 units. In Jimenez’s method, the expected value of the fuzzy parameter can be estimated as the following:

(35) $$EV\left(\tilde{c}\right)=\frac{c^{w}+c^{q}+c^{v}}{4}=\frac{3+2*4+6}{4}=4.25$$

To convert the set of constraints like ${\tilde{a}}_{i}X\geq \tilde{b_{i}},\;i=1,2,\ldots I$, the following convertion is performed:

(36) $$\left(a\times \frac{\left(a_{i}{}^{v}+a_{i}{}^{q}\right)}{2}+\left(1-a\right)\times \frac{\left(a_{i}{}^{w}+a_{i}{}^{q}\right)}{2}\right)X\geq \left(a\times \frac{\left(b_{i}{}^{v}+b_{i}{}^{q}\right)}{2}+\left(1-a\right)\times \frac{\left(b_{i}{}^{w}+b_{i}{}^{q}\right)}{2}\right)$$

If the uncertainty results in the model’s infeasibility, the following set of equations can be accepted:

(37) $$\left(\frac{a}{2}\times \frac{\left(a_{i}{}^{v}+a_{i}{}^{q}\right)}{2}+\left(1-\frac{a}{2}\right)\times \frac{\left(a_{i}{}^{p}+a_{i}{}^{q}\right)}{2}\right)X\geq \left(\frac{a}{2}\times \frac{\left(b_{i}{}^{v}+b_{i}{}^{q}\right)}{2}+\left(1-\frac{a}{2}\right)\times \frac{\left(b_{i}{}^{w}+b_{i}{}^{q}\right)}{2}\right)$$

(38) $$\;\begin{array}{l} \left(\left(1-\frac{a}{2}\right)\times \frac{\left(a_{i}{}^{v}+a_{i}{}^{q}\right)}{2}+\left(\frac{a}{2}\right)\times \frac{\left(a_{i}{}^{w}+a_{i}{}^{q}\right)}{2}\right)X\geq \left(\left(1-\frac{a}{2}\right)\times \frac{\left(b_{i}{}^{v}+b_{i}{}^{q}\right)}{2}+\left(\frac{a}{2}\right)\times \frac{\left(b_{i}{}^{w}+b_{i}{}^{q}\right)}{2}\right) \end{array}$$

Furthermore, when the constraints become non-fuzzy, the membership function proposed by Torabi–Hassani (TH) in [37] is used according to Equation (38):

(39) $$\mu_{F}=\left\{ \begin{array}{ll} 1 & if\;Z<Z^{a-PIS}\\ \frac{Z^{a-NIS}-Z}{Z^{a-NIS}-Z^{a-PIS}} & if\;Z^{a-PIS}\leq Z\leq Z^{a-NIS}\\ 0 & if\;Z>Z^{a-NIS} \end{array}\right.$$

Moreover, when we intend to minimize the objective function, the following membership function is employed:

(40) $$\mu_{F}=\left\{ \begin{array}{ll} 1 & if\;Z>Z^{a-PIS}\\ \frac{Z-Z^{a-NIS}}{Z^{a-PIS}-Z^{a-NIS}} & if\;Z^{a-NIS}\leq Z\leq Z^{a-PIS}\\ 0 & if\;Z<Z^{a-NIS} \end{array}\right.$$

where the positive ideal solutions (*a-PIS*) and the negative ones (*a-NIS*) of the objective function (Z) at the feasibility level (*a*) are considered for the total cost’s objective function. Thus, the fuzzy model equivalent to the original problem’s model can be formulated as follows:

**Problem’s model:**

(41) $$\begin{array}{l} Min\;\;z_{1}=\sum_{r\in R}\sum_{c\in C}\sum_{t\in T}\frac{prm^{w}{}_{pmt}+2prm^{q}{}_{pmt}+prm^{v}{}_{pmt}}{4}\times g_{pmct}\\ +\sum_{f\in F}\sum_{c\in C}\sum_{t\in T}\frac{prf^{w}{}_{pft}+2prf^{q}{}_{pft}+prf^{v}{}_{pft}}{4}\times Xf_{pfct}+\\ \sum_{j\in J}fcj_{j2}\times w_{j2}+\sum_{s\in S}fcs_{s}\times v_{s}+\sum_{b\in B}fcb_{b}\times z_{b}+\sum_{i\in I}\sum_{o\in O}\sum_{t\in T}cpc_{o}\times Xh_{oit^{\prime }}+\\ CTV\times (\sum_{i\in I}\sum_{j\in J}\sum_{t\in T}disa_{ij}\times Xa_{ijt}+\sum_{j\in J}\sum_{f\in F}\sum_{t\in T}disb_{jf}\times Xb_{jft}+\sum_{f\in F}\sum_{t\in T}\sum_{t\in T}disc_{fv}\times Xc_{fpt}\\ +\sum_{f\in F}\sum_{k\in K}\sum_{t\in T}disd_{fk}\times Xd_{fkt}+\sum_{p\in P}\sum_{f\in F}\sum_{m\in M}\sum_{t\in T}dise_{fm}\times Xe_{pfmt}+\sum_{f\in F}\sum_{c\in C}\sum_{t\in T}dis_{fc}\times Xf_{fct}\\ +\sum_{p\in P}\sum_{m\in M}\sum_{c\in C}\sum_{t\in T}disg_{mc}\times Xg_{pmct}+\sum_{o\in O}\sum_{i\in I}\sum_{t\in T}dish_{oi}\times Xh_{oit}+\sum_{o\in O}\sum_{i\in I}\sum_{t\in T}disi_{oi}\times Xi_{oit}+\\ \sum_{b\in B}\sum_{i\in I}\sum_{t\in T}disj_{ib}\times Xj_{ibt}+\sum_{f\in F}\sum_{b\in B}\sum_{t\in T}disk_{fb}\times Xk_{fbt})+\sum_{i\in I}\sum_{t^{\prime }\in T}cpa_{i}\times Xq_{it^{\prime }}+\sum_{j\in J}\sum_{t\in T}chj_{jt}\times Xhj_{jt}\\ +\sum_{p\in P}\sum_{r\in R}\sum_{f\in F}\sum_{t\in T}\left[\frac{cpt^{w}{}_{r}+2cpt^{q}{}_{r}+cpt^{v}{}_{r}}{4}\right]\times Xrh_{fprt}+\sum_{r\in R}\sum_{t\in T}\left[\frac{ctax^{w}{}_{r}+2ctax^{q}{}_{r}+ctax^{v}{}_{r}}{4}\right]\times XSe_{rt} \end{array}$$

Equations (2) and (3)

(42) $$Xq_{it^{\prime }}\leq \left(\frac{\alpha }{2}\times \frac{cap^{v}{}_{it^{\prime }}\;\;+cap^{q}{}_{it^{\prime }}\;\;}{2}+\left(\frac{1-\alpha }{2}\right)\times \frac{cap^{w}{}_{it^{\prime }}\;\;+cap^{q}{}_{it^{\prime }}\;\;}{2}\;\right)\;\;\forall i\in I,t^{\prime }\;\in T$$

Equations (5)–(8)

(43) $$\underset{f\in F}{\sum }{\mathrm{Xe}}_{\mathrm{pfct}}\geq \left(\frac{\alpha }{2}\times \frac{{\mathrm{dco}}_{\mathrm{pct}}{}^{q}+{\mathrm{dco}}_{\mathrm{pct}}{}^{v}}{2}+\left(\frac{1-\alpha }{2}\right)\times \frac{{\mathrm{dco}}_{\mathrm{pct}}{}^{w}+{\mathrm{dco}}_{\mathrm{pct}}{}^{q}}{2}\right)\;\;\;\;\forall c\in C,t\;\in T,\;p\;\in P\;\;\;\;\;\;$$

(44) $$\underset{m\in M}{\sum }{\mathrm{Xg}}_{\mathrm{pmct}}\geq \left(\frac{\alpha }{2}\times \frac{{\mathrm{dct}}^{v}{}_{\mathrm{pct}}+{\mathrm{dct}}^{q}{}_{\mathrm{pct}}}{2}+\left(\frac{1-\alpha }{2}\right)\times \frac{{\mathrm{dct}}^{w}{}_{\mathrm{pct}}+{\mathrm{dct}}^{q}{}_{\mathrm{pct}}\;}{2}\right)\;\;\;\;\;\forall c\in C,t\;\in T,\;p\;\in P\;\;\;\;\;\;\;\;\;$$

Equations (11) and (33).

## 4. Solution Approach

In the current study, a multi-objective MILP model is proposed to create a balance among the cost, the quantity of the pollutants, and the job opportunities in a rice logistics network. In order to solve this model, four metaheuristic algorithms, including a multi-objective version of the reptile search optimizing algorithm called MORSO and three popular algorithms, namely MOSA, MOPSO, and MOGWO, with priority-based encoding are used as the proposed approach. Moreover, the LP-metric method is applied to evaluate the performance of the aforementioned algorithms.

The algorithms of MOPSO and MOSA have been further explained in [21] and MOGWO has been given in [38]. Therefore, the explanation of these algorithms is taken for granted and the MORSO algorithm is described separately in a sub-section. In this section, we explain the proposed solution algorithm in the sub-section Encoding and Decoding and indicate how to satisfy the model’s constraints using this approach. Then, in the continuation, the criteria for comparing the algorithms are described.

### 4.1. Encoding and Decoding

**Figure 10:**
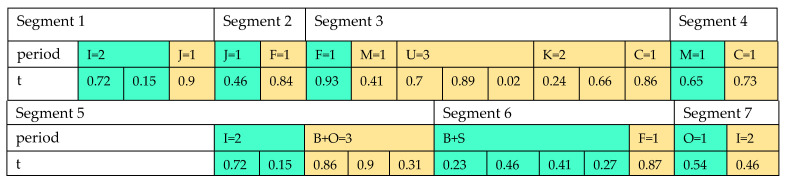
Schematic design of proposed arrays.

In this research, the priority-based encoding method developed by [39] is used to display the initial solution. Considering the encoding and decoding design and method, a small size example is needed to meet the constraints. Here, in Figure 10, the initial solution’s structure is illustrated.

**Figure 11:**
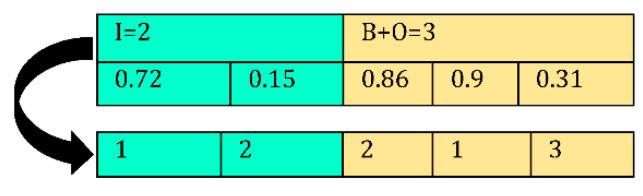
Sorting random numbers.

Suppose that the number of the producers, the distribution centers, the rice factories, the pharmaceutical industries, the toiletry industries, the markets, the customers, the solar panel sites, the bio-refineries, and the recycling centers are 2, 1, 1, 3, 1, 2, 1, 2, 2, and 1, respectively. The proposed array is a matrix with six rows and with the number of columns as 3 ∗ i+2 ∗ j+3 ∗ f+u+k+2 ∗ m+2 ∗ b+2 ∗ o+u. The cells of the 1st sub-segment are filled with random numbers between 0 and 1. In the next stage, as seen in Figure 11, the cells of the 1st sub-segment are sorted by their priority. Sorting the numbers is performed separately for each segment. Regarding the encoding of the 1st segment, whose steps are displayed in Table 2, Constraints (4)–(6) can be satisfied. Moreover, using the 2nd and 3rd segments’ encoding displayed in Table A1 and Table A2 in Appendix A, Constraints (17) and (18) are satisfied. In addition, the inventory in the distribution centers are controlled by the encoding of the 2nd segment. Other constraints are met similarly by encoding the rest of the segments.

### 4.2. Reptile Search Algorithm

The Reptile Search Optimizer (RSO) proposed by [40] is a population-based algorithm that simulates the hunting mechanisms and social behavior of crocodiles in the wild. The algorithm is inspired by some of the key features of crocodile behavior, including encircling the prey and the coordination of crocodiles during an attack. The mechanisms of encircling and hunting prey are mathematically modeled as follows.

#### 4.2.1. Encircling Phase

In this stage, the search space for finding a better solution is analyzed based on two main strategies including high walking and belly walking. Selecting the strategy depends on the number of iterations so that as long as $t\;\leq \;\frac{T}{4}$, the high walking strategy is selected, and as long as $>\frac{T}{4},\;t\leq \frac{T}{2}$, the belly walking strategy is chosen. To update the crocodiles’ position in the exploration stage in each iteration, the following equations are applied:

(45) $$x_{i,j}\left(t+1\right)=\left\{\begin{array}{l} Best_{j}\left(t\right)-\eta_{i,j}\left(t\right)\times \beta -R_{i,j}\left(t\right)\times rand\;\;\;\;\;\;\;\;\;\;\;\;\;\;\;\;\;\;\;\;\;\;\;t\leq \frac{T}{4}\\ Best_{j}\left(t\right)\times x_{r1,j}\times ES\left(t\right)\times rand\;\;\;\;\;\;\;\;\;\;\;\;\;\;\;t\geq \frac{T}{4}\;and\;t<\frac{T}{2} \end{array}\right\}$$

where $Best_{j}\left(t\right)$ stands for the jth position in the best-obtained solution in iteration *t* and *rand* is the random number between 0 and 1 and *T* is the maximum number of iterations., which is calculated according to Equation (46), is a hunting operator for the position in the ith solution. The parameter *β* is responsible for controlling the exploration accuracy in each iteration, which is equal to 0.1. The reduction function, calculated according to Equation (47), reduces the search space. r1 is a random value between the range [1, *N*], and $x_{r1,j}$ indicates the position of the ith solution. *N* is the number of the solutions. Evolutionary Sense (ES (t)) is also a probability ratio of randomly decreasing values between 2 and −2 throughout the number of iterations, which is calculated using Equation (48).

(46) $$\eta_{i,j}\left(t\right)=Best_{j}\left(t\right)\times P_{i,j}$$

(47) $$R_{i,j}\left(t\right)=\frac{\;\;Best_{j}\left(t\right)-x_{\left(r2,j\right)}}{Best_{j}\left(t\right)+\varepsilon }$$

(48) $$ES\left(t\right)=2\times r3\times \left(1-\frac{1}{T}\right)$$

In Equation (47), ε is a small value and r2 is a random number between [1, N]. In addition, r3 denotes a random integer between and 1. $P_{i,j}$ is the percentage difference between the *j*th position of the best-obtained solution and the *j*th position of the current solution, which is calculated using Equation (49):

(49) $$P_{i,j}=a+\frac{x_{i,j}(t)-M(x_{i})}{Best_{j}(t)\times (UB_{j}-LB_{j})+\varepsilon }$$

where, in Equation (49),$\;M\left(x_{i}\right)$ is the average positions of the ith solution, calculated using Equation (50). $UB_{j}$ and $LB_{j}$ are the upper and lower boundaries of the position, respectively. Finally, α is a parameter, which controls the exploration accuracy during iterations, which is fixed equal to.

(50) $$M(x_{i})=\frac{\sum {}_{j=1}^{n}x_{i,j}(t)}{n}$$

#### 4.2.2. Hunting Simulation

The goal behind this mechanism is to escape being trapped in the optimal local points, which is based on two strategies during hunting, coordination and cooperation. Like the encircling mechanism, selecting the strategy depends on the number of iterations so that when, crocodiles select a hunting coordination strategy; otherwise, the cooperation hunting strategy is selected, which is conditioned by $t>\frac{3T}{4},\;t\leq T$. To update the position of the crocodiles in this phase in each iteration, the following equations are applied:

(51) $$x_{i,j}(t+1)=\left\{\begin{array}{cc} Best_{j}(t)\times P_{i,j}(t)\times rand & t\leq \frac{3T}{4}andt>\frac{T}{2}\\ Best_{j}(t)-\eta_{i,j}(t)\times \varepsilon -R_{i,j}(t)\times rand & t\geq Tandt>\frac{3T}{4} \end{array}\right\}$$

where the values of each parameter are similar to what has been pointed out in the previous part. Consequently, the computational complexity of the proposed RSA is as follows:

O(RSA)=O(N×(T×D+1)), where *T* is the number of iterations, *N* presents the number of used solutions, and *D* presents the solution size. The pseudo-code of the RSO algorithm is depicted in Table A3 in the Appendix A.

### 4.3. Multi-Objective Reptile Search Optimization Algorithm

In this sub-section, a multi-objective version of the RSO known as MORSO is proposed in order to solve the proposed multi-objective model. Like other multi-objective metaheuristic algorithms, we deal with concepts such as archive, grid approach, and leader selection in this paper.

#### 4.3.1. Archive and Grid Approach

Archive is in charge of saving, controlling, and retrieving the optimal achieved Pareto solutions. During each iteration, the position of the search agents is updated based on the mechanism of the RSO algorithm and the new obtained solutions are compared with the archive members. If the new solution dominates one of the archive members, it will substitute with it. The archive has a limited capacity, and to delete a solution, the grid approach is employed to select one of the members in the most crowded part of the archive and set it aside.

#### 4.3.2. Selecting a Leader

**Figure 12:**
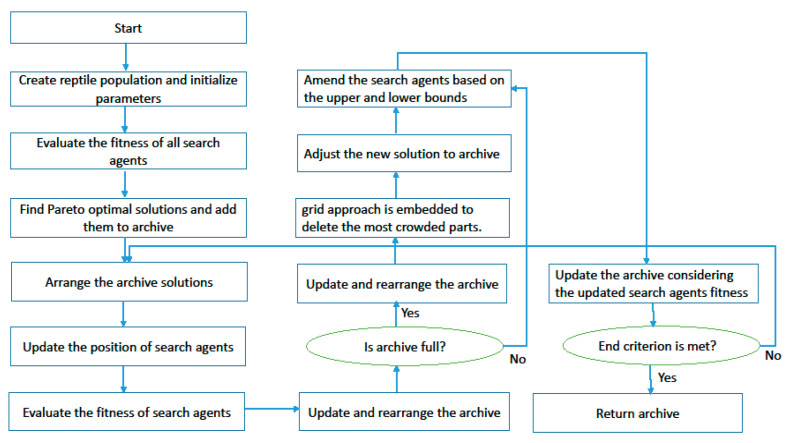
Flowchart of MORSO algorithm.

For selecting a leader, one of the best-obtained optimal solutions in the archive is selected by the roulette wheel method, and other search agents update their position in order to attack the prey. The flowchart and the pseudo-code of the MORSO algorithm are illustrated in Figure 12 and Table 3, respectively.

### 4.4. Evaluation Indices of Algorithms’ Performance

In this research, five indices were used to evaluate the algorithms’ performance. These metrics measure different criteria, which can be listed as follows.

#### 4.4.1. Number of Pareto Solutions (NPS)

In this criterion, the number of Pareto solutions is computed. Every method with a higher NPS criterion is better. The ideal state in this method is that a higher number of Pareto solutions get distributed more evenly in the possible space.

#### 4.4.2. Mean Ideal Distance (MID)

This index is used to calculate the distance between Pareto solutions. Considering Equation (52), the lower this index, the higher the algorithm’s performance. In the current study, the ideal point equals the minimum of each of the objective functions. This index is calculated as shown by Equation (52).

(52) $$\mathrm{MID}=\overset{NOS}{\underset{i=1}{\sum }}\sqrt{\frac{{\left(\frac{f1i-f1best\;}{f1\max total-f1min\;total\;}\right)}^{2}+{\left(\frac{f2i-f2best\;}{f2\max total-f2min\;total}\right)}^{2}}{NOS}}$$

#### 4.4.3. Maximum Spread (MS)

This criterion measures the spread of non-dominated solutions. The more spread out the non-dominated solutions are, the larger this index will be, and the higher this value, the more appropriate. This criterion is estimated by Equation (53) in which *f_j_^max^* and *f_j_^min^* are the max and min values of the objective function _j_ among the non-dominated solutions.

(53) $$\mathrm{MS}=\sqrt{\sum_{i=1}^{I}{\left(Min\;F_{i}-Max\;F_{i}\right)}^{2}}$$

#### 4.4.4. Spread of Non-Dominance Solutions (SNS)

This index is used to check the variety of non-dominance solutions and is calculated by Equation (54). The higher this index, the better the algorithm performs. In this equation, *F*1_*i*_, *F*2_*i*_, and *F*3_*i*_ are the values of the 1st, 2nd, 3rd objective functions for the non-dominance ith solution.

(54) $$\mathrm{SNS}=\sqrt{\frac{\sum_{i=1}^{I}{\left(M-C_{i}\right)}^{2}}{n-1}}\;\;$$

where $C_{i}=\sqrt{F1_{i}{}^{2}+F2_{i}{}^{2}+F3_{i}{}^{2}}$, $M=\frac{\sum {}_{i=1}^{I}\sqrt{F1_{i}{}^{2}+F2_{i}{}^{2}+F3_{i}{}^{2}}}{n-1}$

#### 4.4.5. Computational Time Index (CPU Time)

In large-scale problems, one of the most critical indices is the CPU time, and the lower this value, the lower the efficiency of the algorithm.

## 5. Validation and Analysis of Results

In this section, the accuracy of the programmed model is analyzed by implementing it onto a real case study. Then, in another sub-section, the parameters of the proposed algorithms are tuned to come up with better results. In the third sub-section, to further evaluate the model, sensitivity analysis is performed on some key parameters.

### 5.1. Case Study

**Figure 13:**
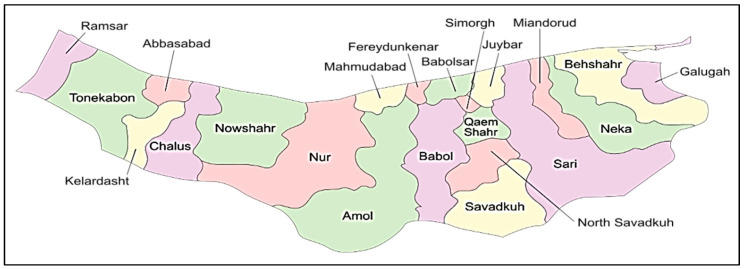
Major cities of Mazandaran Province.

In this section, the accuracy of the programmed model is analyzed by implementing it onto a real case study in Iran, i.e., in Mazandaran Province. At the moment, over 300,000 hectares are under rice cultivation in Iran and this province is the largest producer of rice in Iran [21]. In this study, in order to collect the data, some of the province-based cities were considered as the position of the network facilities; Figure 13 depicts the position of the cities. Moreover, ten sample problems were generated based on the number of the network facilities for evaluating the proposed model’s efficiency, as seen in Table 4. As the information collected from the farmers indicated, the conversion rate of rice plant to paddy is about 0.8. Therefore, the conversion rate of rice to straw is about 0.2. In addition, the conversion rate of paddy to processed rice is about 0.64, and the conversion rates of paddy to bran and to husk are about 0.26 and 0.1, respectively. In bio-refineries, around 26.7 kWh of electricity can be supplied per kg of rice straw and husk. The power generation capacity of solar panel sites ranges from 200 to 400 MWh.

The transportation costs among the facilities correspond with the distance, which is provided in Table A4 in Appendix A. In addition, a nine-ton truck is used to transport the products. This vehicle uses R = 0.0832 fuel per k of transportation and its fuel-emitted CO_2_ rate (φ) is 3.15. The values of these two parameters have been extracted from [38]. Some data are also generated randomly, as demonstrated in Table A5 in Appendix A.

### 5.2. Results Analysis

Firstly the proposed algorithms are tuned to achieve better results. After that, the results from solving the model are analyzed, and for further model evaluation, sensitivity analysis is performed on parameters such as the energy supply capacity and customers’ demand.

### 5.3. The Tune of the Algorithm’s Parameters

There are several methods for tuning the metaheuristic algorithms’ parameters with the goal of improving their performance, out of which the Taghuchi method is utilized in the present paper. Taguchi developed new statistical concepts and combined and established particular groups of orthogonal arrays to present the tests. This method classifies a group of factors based on orthogonal arrays into two main parts, including control and noise factors, and while maximizing the effect of the control factors, it minimizes that of the noise factors according to the following equation [41].

(55) $$\mathrm{SN}\;=-10\;\log\left(\;\frac{\sum_{i=1}^{n}Y^{2}}{n}\right)\;\;\;\;\;$$

where Y stands for the solution value and *n* is the number of the orthogonal arrays.

Moreover, Equation (56) presents the selected responses in this study. Two main concepts related to the solution are convergence and diversity. The MID metric measures the convergence of the solutions and the variety of Pareto solutions is gained by the MS metric [2].

(56) $$\mathrm{MCOV}=\frac{\mathrm{MID}}{\mathrm{MS}}$$

It is necessary to determine the parameters we need to tune in every algorithm to perform the Taguchi test. In the present study, three levels were considered for all parameters given in Table 5. The MOGWO algorithm has three factors, and MOPSO, MOSA, and MORSO algorithms have four factors. By performing the experiment in Minitab software, L9 orthogonal arrays are proposed.

**Figure 14:**
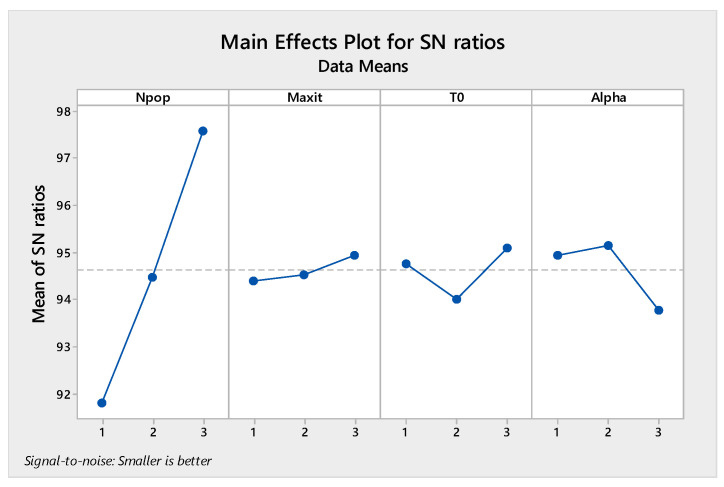
Signal to noise plot of MOSA.

**Figure 15:**
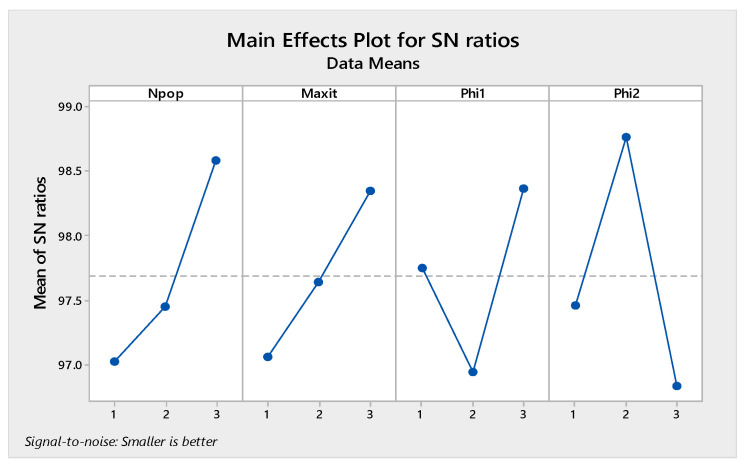
Signal to noise plot of MOPSO.

**Figure 16:**
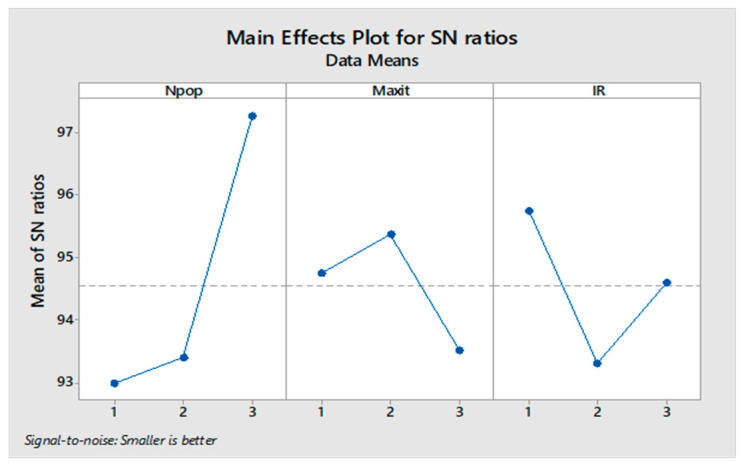
Signal to noise plot of MOGWO.

**Figure 17:**
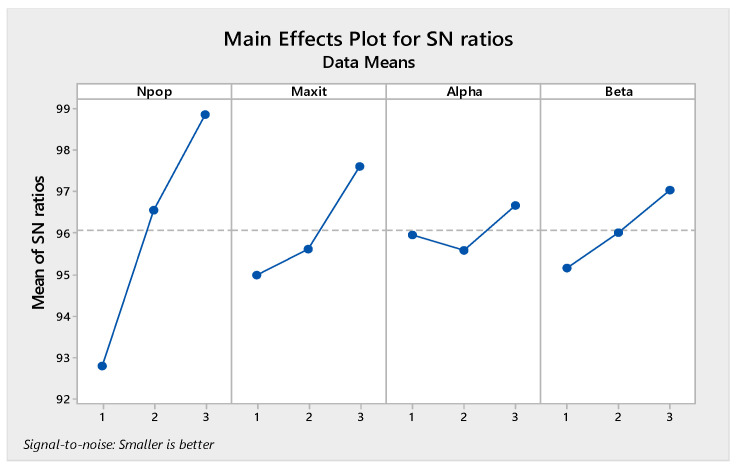
Signal to noise plot of MORSO.

The orthogonal arrays of each algorithm and their derived results are illustrated in Table A6, Table A7, Table A8 and Table A9 in Appendix A. Moreover, the S/N plots for the mentioned algorithms are displayed in Figure 14, Figure 15, Figure 16 and Figure 17, by which the highest level is the best level for each algorithm. As seen in the plots, for example, in the simulated annealing algorithm, the best level for the maximum 3rd level iteration factor is the 3rd one, the best level for the population number factor is the 3rd level, for Phi_1_ factor this is the 2nd level, and for Phi_2_ factor, the best level is the 1st level.

### 5.4. Analysis of Results

We designed 10 sample problems for evaluating the proposed model’s efficiency. For this purpose, a computer with 4 GB of RAM and 2.2 GHz CPU was employed and encoding the proposed models and algorithms was run in MATLAB software. The first to fourth sample problems were solved by LP-metric and the metaheuristic algorithms and the rest were solved by metaheuristic algorithms regarding the model being an NP-hard.

To evaluate the aforementioned metaheuristic algorithms, a one-to-one comparison was performed as the criteria indicated in Section 4. The top performance is based on lower MID and CPU time criteria and higher NPS, MS, and SNS. Table 6 displays the results of this comparison. As seen in the table, metaheuristic algorithms have less execution time compared to the LP-metric method. As the number of sample problems increases, the execution time of the model with the LP-metric method should increase, which indicates that the model is NP-hard. Therefore, we need meta-algorithms in order to solve the model in a reasonable time and reduce its complexity [42,43].

**Figure 18:**
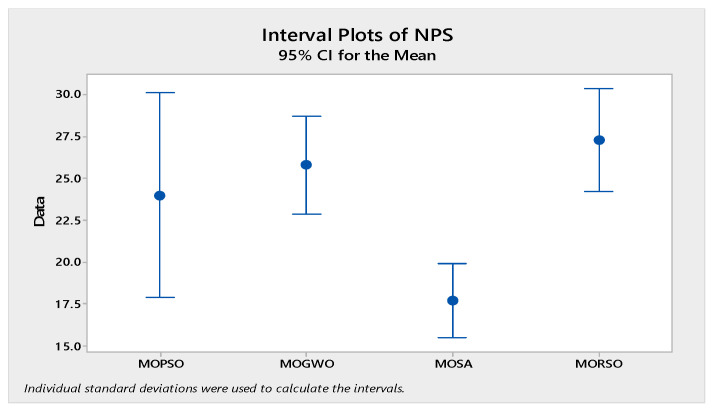
Interval plots of NPS.

**Figure 19:**
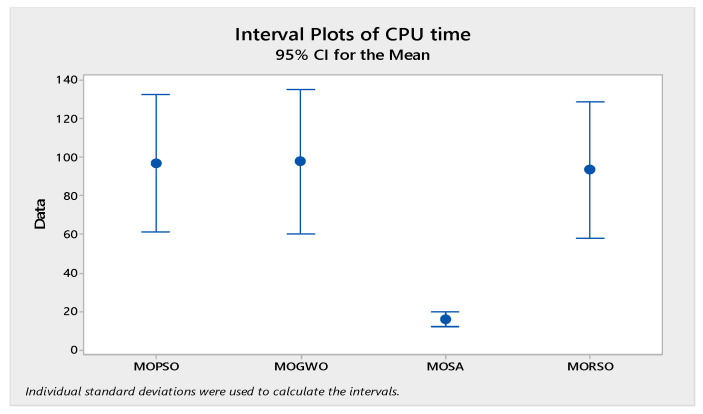
Interval plots of CPU time.

**Figure 20:**
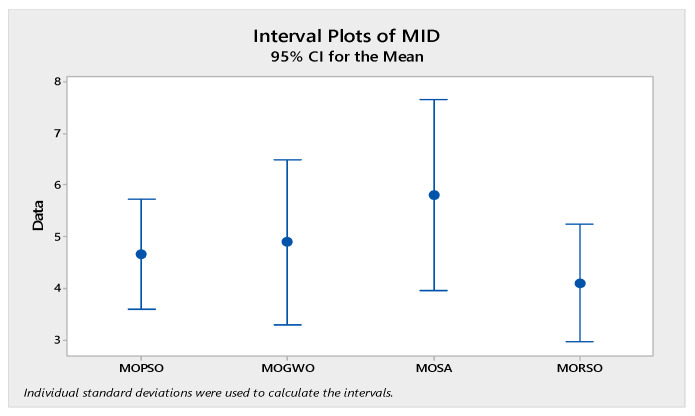
Interval plots of MID.

**Figure 21:**
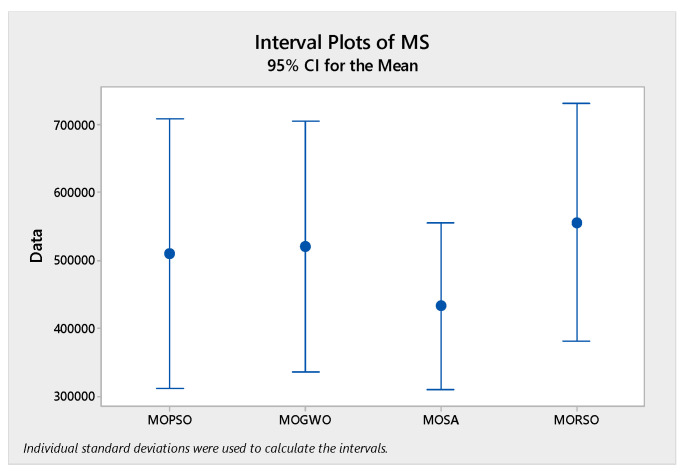
Interval plots of MS.

**Figure 22:**
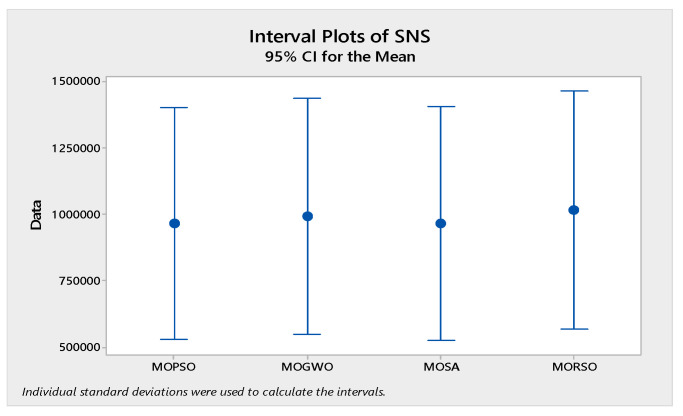
Interval plots of SNS.

**Figure 23:**
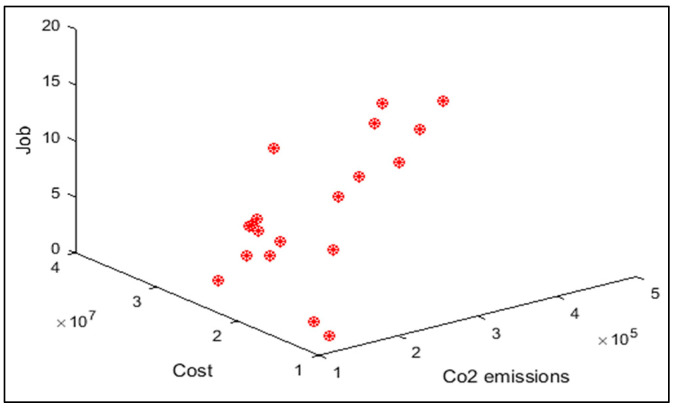
Pareto front of the first test problem from MOGWO.

**Figure 24:**
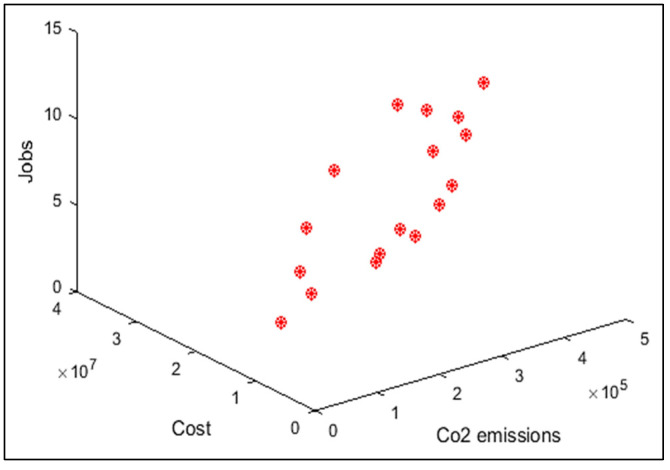
Pareto front of the first test problem from MOSA.

**Figure 25:**
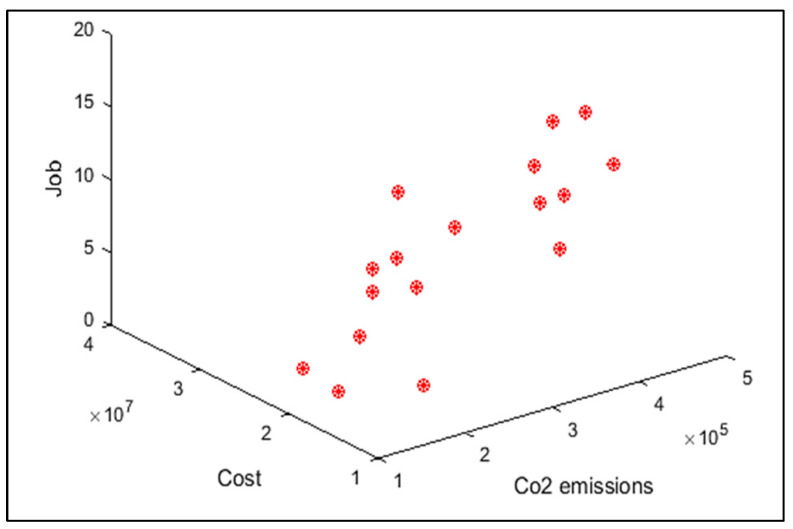
Pareto front of the first test problem from MOPSO.

**Figure 26:**
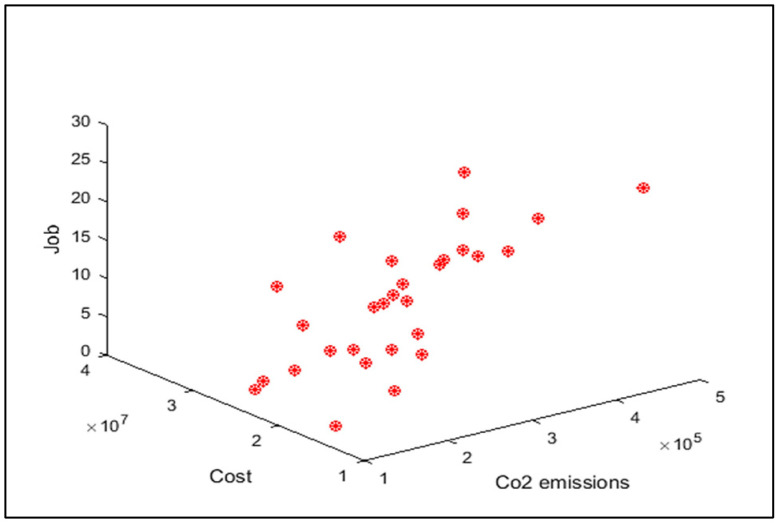
Pareto front of the first test problem from MORSO.

In order to compare the algorithms in terms of performance, ANOVA was used. The intervals plot (at confidence level 95%) are sketched pursuant to the data in Table 6 separately for each of the algorithms and according to each criterion in Figure 18, Figure 19, Figure 20, Figure 21 and Figure 22. As perceived from these plots, the MORSO algorithm performed better than other algorithms in terms of NPS, MS, and MID criteria. These plots indicate that MOSA is faster than other algorithms. In terms of SNS criteria, all three algorithms performed similarly close to each other. Thus, it can be concluded that the MORSO algorithm performed better than other algorithms. An example of non-dominance solutions for the first sample problem for the mentioned metaheuristic algorithm is shown in Figure 23, Figure 24, Figure 25 and Figure 26.

### 5.5. Sensitivity Analysis

In this sub-section, for further evaluation of the proposed model and the top algorithm, the sensitivity analysis is conducted in two states. It is worth mentioning that sensitivity analysis was performed for the first sample problem. In the first state, the sensitivity analysis is performed on the parameter of capacity of each energy source, and in the second state, on the parameter of customers’ demand for the processed rice.

#### 5.5.1. Sensitivity Analysis on Energy Source Capacity Parameter

In this state, it is assumed that the capacity of each energy source would decrease or increase consistent with the conditions listed in Table 7.

**First condition**: It is assumed that all electricity required for product processing should be supplied by the mains electricity and not by the renewable energy sources.

**Second condition**: It is assumed that the capacity of mains electricity would decrease by 20% and the capacity of renewable energy sources would increase by 30%.

**Figure 27:**
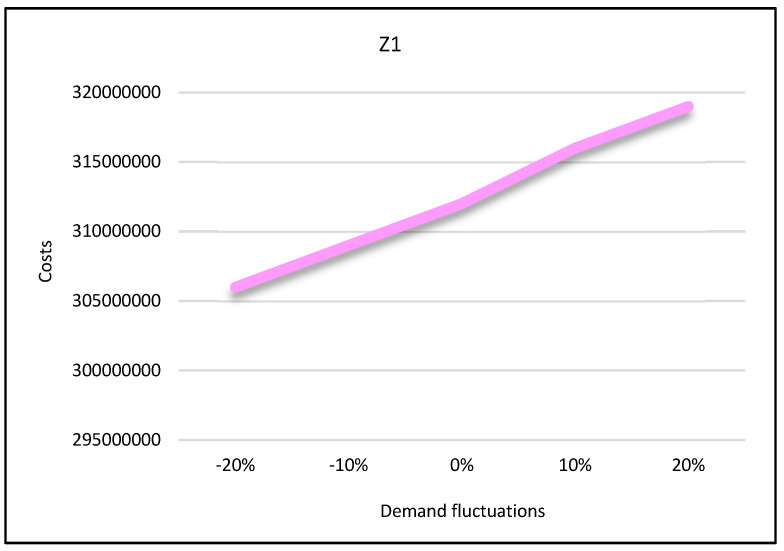
Demand variation effect on first objective function.

**Figure 28:**
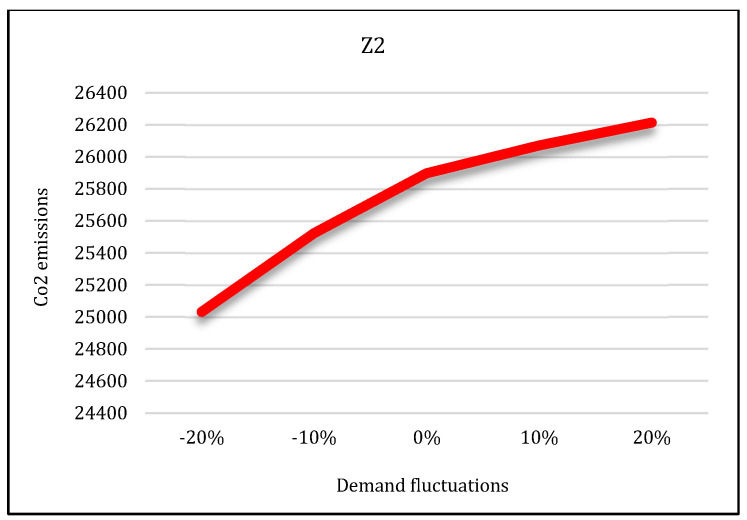
Demand variation effect on second objective function.

**Figure 29:**
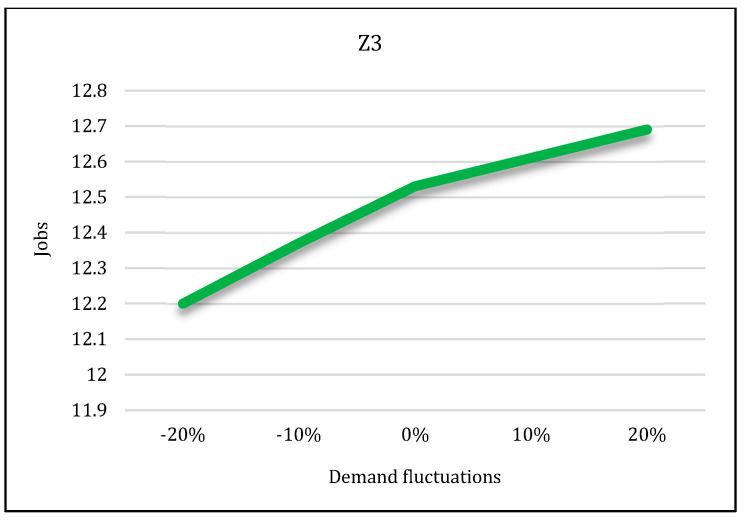
Demand variation effect on third objective function.

**Third condition**: It is assumed that the capacity of the mains electricity would decrease by 30% and the capacity of renewable energy sources would increase by 40%. Having solved the model under these conditions by the LP-metric method, the results are provided in Figure 27, Figure 28 and Figure 29. The first condition states that the utilization of the mains electricity would increase by about 19%. The second and third conditions state that with the renewable sources’ capacity increase, the utilization of mains electricity would decrease by about 7% and 9%, respectively.

#### 5.5.2. Sensitivity Analysis on Demand

In the second state, sensitivity analysis was carried out on the traditional shopping consumers’ demand parameter. Sensitivity analysis is performed under five states in which the demand parameter decreases and increases. Suppose that consumers’ demand increases by 10% and 20% in two states and decreases by the same level in two states. The third state is consistent with the base state. After solving the model under all conditions, the results are displayed in Figure 27, Figure 28 and Figure 29.

According to these figures, as the demand increases, all three objective functions of the model also increase, and vice versa. In other words, if customer demand for rice increases, the costs, pollutants, and job opportunities created in the supply chain network will also increase. Due to the increasing demand for rice, farmers are forced to produce more crops. As a result, production costs and energy consumption taxes will also increase, and, therefore, the first objective function will increase. In addition, more product will be moved between facilities and more pollutants will be produced. Therefore, the second objective function will also increase. Finally, due to the production of more crops, more job opportunities will be created in rice farms. Thus, the third objective function will also increase. Therefore, we can conclude that demand and all three objective functions of the model have a direct relation.

## 6. Conclusions, Managerial Insight, and Future Works

At the present time, rice feeds over half of the world population, which is globally crucial in our food systems. Establishing more efficient and sustainable rice value chains could enable the UN to achieve the Sustainable Development Goal of Zero Hunger by 2050. In order to achieve this goal, it is essential to properly manage supply chains and create a balance between the supply and demand of rice, which can solve many supply problems of this product.

In the present study, a dual-channel CLSC network of a sustainable supply chain was designed for a rice plant while considering energy sources in an uncertain environment. The network included the producers, the distribution centers, the rice factories, the pharmaceutical industries, the toiletry industries, the markets, the customers, the solar panel sites, the bio-refineries, and the recycling centers. The grid also supplied electricity needed to process rice through bio-refineries and solar panel sites. In this network, the required electrical energy for rice processing was supplied through bio-refineries and the solar panel site as well. Regarding how rice waste as biomass has the potential to generate energy, this study addressed this issue and proposed a mathematical model for optimally using rice waste for energy generation.

Then, the total costs, the quantity of the emitted pollutants, and the fixed and variable job opportunities created in the proposed network were optimized via an MILP model under the uncertainty of cost, supply, and demand. The goal of the proposed model was to determine the optimal quantity of rice production and distribution and its waste among the network facilities.

The costs include the total transportation costs among the network facilities, the production costs for the farmers and rice factories, the product maintenance costs for the distribution centers, and the fixed costs for reopening new distribution centers, the bio-refineries, and the solar panel sites. In the present research, the parameters cost, supply, and demand were assumed uncertain, and fuzzy logic was applied to deal with uncertainty. To solve the proposed model, MOPSO, MOSA, and MOGWO algorithms and a new multi-objective version of the Reptile Search Optimizer called MORSO were used. Then, the mentioned algorithms were validated using LP-metric in small-sized samples. Furthermore, their results and performance were compared based on criteria such as MS, SNS, NPS, MID, and CPU time. Moreover, for confirming the model’s validity, ten sample problems in different sizes were designed. In addition, for coming up with the best performance, the parameters of all three algorithms were tuned by the Taguchi method. After solving the model, the derived results were evaluated and these algorithms’ performances were analyzed by ANOVA through the interval plots at the confidence level of 95%. A significant statistical difference was observed in terms of the performances of these algorithms, and according to the statistical tests, the MORSO algorithm performed better than other algorithms in terms of NPS, MS, and MID criteria. Furthermore, for more model evaluation, the sensitivity analysis was performed on the key parameters. The numerical results indicated that it is possible to save up to 19% of electricity consumption by constructing the solar panel sites and producing energy from the rice waste.

Considering management, the present study’s results can benefit the relevant managers and the countries producing rice. The present study’s findings will be highly useful and involve remarkable applications for decision making on opening solar panel sites and bio-refineries for supplying the required electrical energy for rice processing through focusing on the sustainable dimensions. The related results and findings have been provided. The findings of this research in the areas of clean energy consumption and job opportunities can be stated as follows:

- **Job opportunity**: Agricultural growth has led to increasing the productivity and income of small and marginal farmers and raising the employment and wages of workers. Consequently, it is critical to consider this dimension of sustainability in supply chain optimization. The proposed model pursues the goal of raising job opportunities and its results revealed that increasing rice production could boost the intended goal.
- **Clean energy consumption**: Using solar energy as a source of clean energy has gained importance considering the non-renewable nature of fossil sources such as oil and gas. Solar energy and biomass are viewed as a source of clean energy and the cost of generating electricity by them is less than that generated by fossil fuels. Moreover, they emit less pollution and fewer greenhouse gases. Regarding how rice straw and husk have the potential to generate energy, the current research has dealt with this matter and a mathematical model has been formulated to make strategic decisions about the construction of solar panel sites and bio-refineries and optimally using rice waste for energy generation.

### Limitations of the Current Study

The current research has many limitations and it seems that different directions can be considered for its development. To develop this study in the future, different methods of rice production and the capacity of energy sources could be considered. Moreover, water resources are of crucial importance in rice production, thus sustainability should be promoted by considering water resources issues in future works. Moreover, considering other uncertainty approaches such as stochastic, probability, and possibilistic is one of the issues that could be emphasized by the researchers in this field and could be incorporated to improve the model. Furthermore, solving the proposed model by the heuristic methods and other metaheuristic algorithms and comparing their results might be interesting. Finally, integrating the proposed model with topics such as the Internet of Things and Industry 4.0 could also be considered by researchers. 

## Figures and Tables

**Table 1:** Some papers published in the scope of ASC.

Authors	Model	Objectives	Uncertainty	Period		Online Purchase	Energy Source	Case Study	Solution Method
				Single	Multi				
[15]	LP	Minimizing the transportation costs		*				Fruit	Exact
[29]	LP	Minimizing costs Minimizing water consumption, CO_2_ emissions, and destructed jobs			*			An agro-food company	AHP
[18]	MILP	Minimizing total cost Maximizing demand responsiveness			*			Citrus	Meta-heuristics
[21]	MILP	Minimizing total cost			*			Rice	Meta-heuristics
[19]	MILP	Minimizing total cost	Robust		*			Wheat	Exact
[2]	MILP	Minimizing CO_2_ emissions Minimizing total cost Maximizing demand responsiveness			*			Citrus	Meta-heuristics
[34]	MILP	Minimizing total cost		*				Wheat	Benders decomposition
[32]	MILP	Minimizing total cost Minimizing water consumption	Simulation		*			Wheat	Goal programming
[34]	MILP	Minimizing total cost		*				Wheat	Benders decomposition
[33]	MILP	Maximizing total profit Minimizing CO_2_ emissions	Roust fuzzy		*			Pistachio	Epsilon constraint
[35]	MILP	Minimizing total cost			*			Crops	Lingo
[22]	MILP	Minimizing total cost			*			Walnut	Meta-heuristics
[24]	MILP	Minimizing total cost			*			Sugarcane	Meta-heuristics
[23]	MILP	Minimizing total cost Maximizing the created jobs			*			Avocado	Meta-heuristics and Exact
The present study	MILP	Minimizing total cost Maximizing the created jobs Minimizing CO_2_ emissions	Fuzzy		*	*	*	Rice	Meta-heuristics and Exact

The asterisk means the model is single period or multi period.

**Table 2:** Proposed priority-based decoding procedure of segment 1.

***For**t* = 1:*T*

***Inputs:*** *I* = set of producers *J* = set of distribution centers
Ca(i,t) = production capacity of producer i in period *t*
D(j,t)= capacity of distribution center j in period t
V(L+N)= encode solution of period t
Dis(i,j) = Distance between nodes

***Outputs:***
Xaloc(i,j,t)= amount of shipments between node i and j in period t
Em(i,j,t)=$\mathrm{Amount}\;\mathrm{of}\;{\mathrm{CO}}_{2}\;\mathrm{emission}\;\mathrm{caused}\;\mathrm{by}\;\mathrm{transferring}\;\mathrm{quantity}\;\mathrm{between}\;\mathrm{node}\;i$ and in period *t*
W(j) = binary variable shows the distribution centers *j* is opened
Step1=Xaloc(i,j,t)=0 i∈I, j∈J $while\underset{i}{\sum }Ca\left(i,t\right)>0\;\;\;or\;\;\underset{j}{\sum }D\left(j,t\right)>0\;\;\;\;\;\;\;\;\;\;\;\;\;\;\;\;\;\;\;\;\;\;$
**Step3** = Xaloc(i,j,t)=min(Ca(i,t), Cap(j,t))
Update demands and capacities
Ca(i,t)=Ca(i,t)−Xaloc(i,j,t), D(j,t)=D(j,t)−Xaloc(i,j,t)
**Step4 =** if Ca(i,t)=0 then V(I,J)=0; if D(j,t)=0 then V(I,J)=0;
End while **Step5 =** $Em{\left(i,j,t\right)}^{\;}=\left(Xaloc\left(i,j,t\right)/Cap_{v}\times RF_{f}\times Dis_{ij}\right)$
For i∈I **If** $\sum_{i}Xaloc(i,j,t)>0thenW(j)=1$
***End if*** ***End for*** ***End for***

**Table 3:** Pseudo-code of MORSO algorithm.

***Input*:** Reptile population and parameters
***Output*:** Archive of non-dominated optimal solutions
Calculate the fitness value of each search agent,
Determine the non-dominated reptiles and add them to archive
***While*** (t < Maxiteration) ***do***
***For*** each search agent ***do***
Update the position of current search agent based on RSO mechanism
***End for***
Compute the fitness of all search agents
Find the non-dominated optimal solutions from updated search agents
Update the obtained non-dominated reptiles to archive
***If*** archive becomes full ***then***
Check if any search agent goes beyond the search space and then adjust it
Compute the objective function values of each search agent
*t* = *t* + 1
***End while***
Return archive

**Table 4:** General data of test problem.

Test	*I*	*J*1	*J*2	*F*	*M*	*K*	*O*1	*O*2	*V*	*B*	*U*
1	6	2	1	2	2	1	3	1	2	1	2
2	13	4	2	4	3	2	5	2	4	3	3
3	22	7	3	8	8	2	7	4	6	6	8
4	30	9	5	9	6	3	9	7	9	8	11
5	40	15	7	14	9	5	14	11	11	12	14
6	55	20	8	20	14	9	23	13	18	17	17
7	65	24	11	24	16	12	28	15	25	24	22
8	72	30	14	28	20	15	35	16	31	30	26
9	80	34	17	32	24	19	42	18	37	39	34
10	82	38	20	36	28	21	49	20	42	40	39

**Table 5:** Applied algorithms’ parameter levels and their values.

Algorithm	Parameter	Parameter Level			Best Level
		Level 1	Level 2	Level 3	
MOSA	Maximum iteration (Maxiter)	50	100	150	150
	Population size (Npop)	40	50	60	60
	T	30	40	50	50
	Alpha	0.9	0.95	0.99	0.95
MOGWO	Maximum iteration (Maxiter)	50	100	150	150
	Population size (Npop)	40	50	60	50
	Initialization ratio (IR)	0.5	0.6	0.7	0.5
MOPSO	Maximum iteration (Maxiter)	50	100	150	150
	Population size (Npop)	40	50	60	50
	C_1_	1.9	2	2.1	2.1
	C_2_	2	2.1	2.2	2.1
MORSO	Maximum iteration (Maxiter)	50	100	150	150
	Population size (Npop)	40	50	60	60
	Alpha	0.1	0.12	0.14	0.1
	Beta	0.1	0.12	0.14	0.12

**Table 6:** Evaluation of mentioned algorithms in each metric measure.

Problem	NPS					CPU Time (Second)				
	MOPSO	MOGWO	MOSA	MORSO	LP-Metric	MOPSO	MOGWO	MOSA	MORSO	LP-Metric
1	22	24	16	25	11	29	28	4.1	25	1223
2	13	22	14	24	11	44	40	3.3	37	1757
3	23	24	15	24	12	57	54	3.6	53	2598
4	36	35	17	37	11	67	71	4	68	3150
5	26	24	23	28	13	83	88	4.2	85	5294
6	28	30	16	32	NA	98	101	4.5	96	NA
7	17	21	15	23	NA	116	125	4.8	112	NA
8	32	27	20	27	NA	137	139	5.1	132	NA
9	29	25	21	27	NA	155	163	5.4	151	NA
10	27	26	20	29	NA	179	197	5.6	173	NA
**Problem**	**MS**					**SNS**				
	**MOPSO**	**MOGWO**	**MOSA**	**MORSO**	**LP-Metric**	**MOPSO**	**MOGWO**	**MOSA**	**MORSO**	**LP-Metric**
1	76,996	78,902	80,928	81,616	813,421	97,270	76,686	62,796	98,209	96,036
2	179,312	191,723	329,516	258,032	293,404	322,501	303,975	300,817	331,716	339,023
3	362,921	402,387	334,579	410,477	425,328	476,055	493,922	475,192	498,241	500,445
4	483,448	512,464	415,642	517,116	505,373	637,523	682,038	598,916	672,870	675,803
5	495,925	472,086	426,705	522,802	480,042	773,715	876,200	834,830	881,936	852,516
6	514,229	503,311	350,392	530,459	NA	1021,995	1031,169	1087,758	1105,495	NA
7	668,699	613,566	597,331	608,137	NA	1241,442	1262,644	1241,226	1293,651	NA
8	348,317	563,723	552,739	583,602	NA	1489,544	1549,389	1438,068	1567,032	NA
9	737,518	794,779	649,699	774,415	NA	1664,923	1723,552	1732,838	1743,955	NA
10	835,031	887,315	587,273	906,189	NA	1947,220	1931,833	1895,988	1963,370	NA
**Problem**	**MS**									
	**MOPSO**	**MOGWO**	**MOSA**	**MORSO**	**LP-Metric**					
1	1.56	1.3	1.5	1.2	1.3					
2	4	2.5	3.7	3.1	2.2					
3	3.5	3.2	2.5	2.3	2.46					
4	4.2	3.7	5.4	3.5	3.1					
5	4.7	6.1	5.2	4.4	4.2					
6	4.8	4.6	6.5	4.3	NA					
7	5.4	6.9	8.8	5.5	NA					
8	7.1	4.9	8.6	4.7	NA					
9	5.4	7.7	8	5.9	NA					
10	5.9	7.9	7.8	6.1	NA					

**Table 7:** Energy sources capacity variation effect.

	*Capr_rt_*			*Xse_rt_*		
Condition	Bio-Refinery	Solar Panels	Mains Electricity	Bio-Refinery	Solar Panels	Mains Electricity
1	0	0	+19%	−100%	−100%	-
2	+7%	13%	−7%	+30%	+30%	−30%
3	8%	16%	−9%	+50%	+50%	−40%

## Data Availability

Data available on request authors.

## Abbreviations

**Sets and Indicators:**	
*I*	Set of producers i∈I
*J*_1_	Set of existing distribution centers $j_{1}\in J_{1}$
$J_{2}$	Set of new distribution centers $j_{2}\in J_{2}$
*J*	$\mathrm{Set}\;\mathrm{of}\;\mathrm{all}\;\mathrm{distribution}\;\mathrm{centers}\;j\in J=J_{1}$⋃$\;J_{2}$
*V*	Set of pharmaceutical industries v∈V
*F*	Set of rice factories f∈F
*K*	Set of toiletries industries k∈K
*M*	Set of retailers (market) m∈M
*O*_1_	$\mathrm{Set}\;\mathrm{of}\;\mathrm{existing}\;\mathrm{recycling}\;\mathrm{centers}\;o_{1}\in O_{1}$
*O*_2_	$\mathrm{Set}\;\mathrm{of}\;\mathrm{new}\;\mathrm{recycling}\;\mathrm{centers}\;o_{2}\in O_{2}$
*O*	Set of all recycling centers *o* ∈ *O* = *O*_1_ *⋃* *O*_2_
*C*	Set of customers *c* ∈ *C*
T	$\mathrm{Set}\;\mathrm{of}\;\mathrm{time}\;\mathrm{period}\;t\in T=\left\{1,2,\ldots \;t^{\prime },\ldots ,t\right\}$
*T′*	$\mathrm{Index}\;\mathrm{for}\;\mathrm{harvest}\;\mathrm{period}\;t^{\prime }\in T^{\prime }=\left\{1,2\right\}$
*U*	Set of solar panel sites *u* ∈ *U*
*B*	Set of bio-refineries sites *b* ∈ *B*
*X*	Indicator for processed rice
*Y*	Indicator for rice bran
*P*	Set of products p∈{X,Y}
*G*	Indicator for mains electricity
*R*	Set of energy sources r∈R={ mains electricity, solarpanel, bio-refinery}
**Parameters:**	
$fcj_{j}{}_{2}$	Fixed cost for launching new distribution center *j_2_*
$fcs_{s}$	Fixed cost for launching solar panel site s
$fco_{o2}$	Fixed cost for launching recycling distribution center o_2_
$fcb_{b}$	Fixed cost for launching bio-refinery *b*
${\tilde{prm}}_{pmt}$	Cost of purchasing each kg of p-type product from market m (traditional purchase) during period *t*
${\tilde{prf}}_{pft}$	Cost of purchasing each kg of p-type product from factory f (online shopping) during period *t*
$pcapf_{pft}$	Production capacity of p-type product in factory f during period *t*
$cpa_{i}$	Cost of production per kg of rice plant for farmer *i*
$\tilde{cta}x_{r}$	Tax on consumption per unit of electricity supplied from source *r*
$\tilde{cp}t_{pfr}$	Production cost per kg of p-type product in factory *f*, whose electricity is supplied from source *r*
$chj_{j}{}_{t}$	Cost of per kg unprocessed rice maintenance at distribution center *j* during period *t*
CT	Cost of transporting by vehicle per kilometer
$disa_{ij}$	Distance between producer *i* and distribution center *j*
$disb_{jf}$	Distance between distribution center j and rice factory *f*
$disc_{fv}$	Distance between rice factory *f* and pharmaceutical industry *v*
$disd_{fk}$	Distance between rice factory *f* and toiletries industry *k*
$dise_{fm}$	Distance between rice factory f and market *m*
$disf_{fc}$	Distance between rice factory *f* and customer *c*
$disg_{mc}$	Distance between market *m* and customer *c*
$dish_{io}$	Distance between producer *i* and recycling center *o*
$disi_{oi}$	Distance between recycling center *o* and producer *i*
$disj_{ib}$	Distance between producer *i* and bio-refinery *b*
$disk_{fb}$	Distance between rice factory *f* and bio-refinery *b*
$cap_{it^{\prime }}$	Product’s production capacity by producer *i* during period *t′*
$capr_{rt}$	Max energy level supplied from source *r* during period *t*
${\tilde{dct}}_{pct}$	Demand for p-type product by consumer *c* in traditional purchase during period *t*
${\tilde{dco}}_{pct}$	Demand for p-type product by consumer *c* in online shopping during period *t*
$dci_{it}$	Demand for compost by farm *i* during period *t*
$dv_{vt}$	Demand for rice bran by pharmaceutical industry *v* during period *t*
$dk_{kt}$	Demand for rice bran by toiletries industry *k* during period *t*
ER	Electrical energy required to process per kg of rice
*M*	A very large positive number
$\eta_{\;}$	Paddy to husk conversion rate
δ	Rice husk to electrical energy conversion rate in bio-refineries
λ	Straw to electrical energy conversion rate in bio-refineries
ψ	Straw to compost conversion rate
$cpo_{o}$	Cost of production per kg of compost at recycling center *o*
$\pi o_{o}$	CO_2_ quantity released from constructing recycling center *o*
$\pi s_{s}$	CO_2_ quantity released from constructing solar panel *s*
$\pi b_{b}$	CO_2_ quantity released from constructing bio-refinery *b*
*RF*	Fuel required for vehicle per travel per *k*
capv	Capacity of vehicle
β	Rice plant to paddy conversion rate
$1-\beta_{\;}$	Rice plant to straw conversion rate
$\theta_{p}$	Paddy to product of type-p conversion rate
$fj_{j2}$	Number of fixed job opportunities created by constructing new distribution centers *j_2_*
$VJI_{i}$	Number of variable job opportunities created by producing per kg of product on farm *i*
$VJQ_{pf}$	Number of fixed job opportunities created by producing per kg of product p in factory *f*
**Decision Variables**	
$XSe_{rt}$	Amount of electrical energy that should be supplied from source *r* during period *t*
$Xhj_{jt}$	Quantity of paddy rice stored in the distribution center j during period *t*
$Xa_{ijt^{\prime }}$	Quantity of rice plant transferred from producer *i* to distribution center *j* during period *t′*
$Xb_{jft\;}$	Quantity of paddy rice transferred from distribution center *j* to rice factory *f* during period *t*
$Xc_{fvt\;}$	Quantity of rice bran transferred from rice factory *f* to pharmaceutical industry *v* during period *t*
$Xd_{fkt}$	Quantity of rice bran transferred from rice factory f to toiletries industry *k* during period *t*
$Xe_{pfmt}$	Quantity of type-p product transferred from rice factory *f* to market *m* during period *t*
$Xf_{pfct}$	Quantity of type-p product transferred from rice factory *f* to customer *c* during period *t*
$Xg_{pmct}$	Quantity of type-p product transferred from market *r* to customer *c* during period *t*
$Xh_{iot^{\prime }}$	Quantity of compost transferred from producer *i* to recycling center *o* during period *t′*
$Xi_{oit}$	Quantity of compost transferred from recycling center *o* to producer *i* during period *t*
$Xj_{ibt}$	Quantity of rice straw transferred from farm *i* to bio-refinery *b* during period *t*
$Xk_{fbt}$	Quantity of rice bran transferred from rice factory *f* to bio-refinery *b* during period *t*
$Xq_{it^{\prime }}$	Amount of rice plant produced by producer *i* during period *t′*
$Xrh_{fprt}$	Quantity of type-p product produced from rice factory f using source *r* during period *t*
$XSe_{rt}$	Quantity of electrical energy that should be supplied from source *r* during period *t*
**Variables Zero and One:**	
*W_j_*	if distribution center *j* is established, 1
*V_s_*	if solar panel site *s* is established, 1
*Z_b_*	if bio-refinery *b* is established, 1
*Q_o_*	if recycling center *o* is established, 1

## Appendix A

**Table A1:** The proposed priority-based decoding procedure of segment 2.

***For*** *t* = 1 to *T* ***Inputs*** *J* = set of distribution centers
*F* = set of rice factories
D(f,t) = capacity of rice factories f in period t
Ca(j,t)= capacity of distribution center j in period t
*V(J + F)* = encode solution of period t
Dis(j,f): distance between nodes
--------------------------------------------------------------------------------------
***Outputs***:
yaloc(j,f,t) = amount of shipments between node j and f in period t
Em(j,f,t)= amount of CO_2_ emission caused by transferring quantity
by vehicle between nodes
INV(j,t) = amount of remained goods in distribution center j at period t
-------------------------------------------
Ca(j,t)=Ca(j,t)+INV(j,t−1)
step1=yaloc(j,f,t)=0 j∈J, f∈F

***while*** ∑_i_*Ca*(*j*,*t*) > 0 or ∑*_i_D*(*f*,*t*) > 0
step2: select the value of first column of first sub-segment *J* for *j* index)
select the value of first column of first sub-segment *F* for *f* index
step3: yaloc(j,f,t)=min(Ca(j,t), D(f,t))
Update demands and capacities
Ca(j,t) =Ca(j,t) −yaloc(j,f,t) , D(f,t)=D(f,t)−yaloc(j,f,t)
step4:if Ca(j,t)=0 then V(J,F)=0; ***if*** D(f,t)=0 then V(J,F)=0;
***End while***
$\mathrm{step}5:Em{\left(j,f,t\right)}^{\;}=\left(yaloc\left(j,f,t\right)/Capv_{\;}\times RF_{\;}\times Dis_{jf}\right)$
step6:INV(w,t)=Ca(w,t) ***End for***

**Table A2:** The proposed priority-based decoding procedure of segment 3.

**For***t* = 1: *T*
***Inputs***: *F* = set of rice factories *P* = set of product
*L* = set of all customers
pcap(p,f,t) = Production capacity of rice factory f for product p in period t
D(p,l,t)= demand of customer l in period t
V(F+L) = Encode solution of period t
Dis(f,l): distance between nodes ------------------------------------------------------------------------------------------------------- ***Outputs***:
zaloc(p,f,l,t)=amount of product p shipments between nodes f and l in period t
Em(f,l,t)= amount of CO_2_ emission caused by transferring quantity
by vehicle between nodes
TK(p,f,t) = number of created job opportunities thorough production of product p in factory f in period t --------------------------------------------------------------------------------------------------------
Step1=zaloc(p,f,l,t)=0 f∈F, l∈L
$while\;\;\;\;\;\sum_{i}pcap\left(p,f,t\right)>0\;\;\;or\;\;\sum_{i}D\left(p,l,t\right)>0\;\;\;\;\;\;\;\;\;\;\;\;\;\;\;\;\;\;\;\;\;\;$
step2: select the value of first column of first sub−segment F for f index
select the value of first column of first sub−segment L for l index
step3: zaloc(p,f,l,t)=min(pcap(p,f,t), D(p,l,t))
Update demands and capacities
pcap(k,t)=pcap(p,f,t)−zaloc(p,f,l.t) , D(p,l,t)=D(p,l,t)−zaloc(p,f,l,t)
step4:if pcap(p,f,t)=0 then V(K,L)=0;
if D(p,l,t)=0 then V(K,L)=0;
***End while***
for p=1
for k=1
$\mathrm{Step}5:Em{\left(f,l,t\right)}^{\;}=\left(zaloc\left(p,f,l,t\right)/{Capv}_{\;}\times {RF}_{\;}\times {Dis}_{fl}\right)$
$\mathrm{Step}6:\mathrm{let}\;Xp\left(p,f,t\right)=\sum_{l\in L}zaloc\left(p,f,l,t\right)\;\mathrm{and}\;TK\left(p,f,t\right)=\frac{Xp\left(p,f,t\right)}{pcap\left(p,f,t\right)}$
***End for***

**Table A3:** Pseudo-code of RSO algorithm.

***Initialize*** RSA parameters *α*, *β*, etc.
***Initialize*** the solutions’ positions randomly. X: i=1, …,N.
***While*** (t < T ) ***do***
Calculate the Fitness Function for the candidate solutions (X).
Find the Best solution so far.
Update the *ES* using Equation (51).
***For*** (i=1 to N) ***do***
***For*** (j=1 to n) ***do***
Update the *η*, R, P and values using Equations (49), (50) and (52), respectively.
***If*** $(t\;\leq \frac{T}{4}$ ) ***then***
$x_{i,j}\left(t+1\right)$= $Best_{j}\left(t\right)-\eta_{i,j}\left(t\right)\times \beta -R_{i,j}\left(t\right)\times randt$
***Else if*** ($randt\geq \frac{T}{4}\;and\;t<\frac{T}{2}$ ) ***then***
$x_{i,j}\left(t+1\right)$= $Best_{j}\left(t\right)\times x_{r1,j}\times ES\left(t\right)\times randt$
***Else if*** ($t>\frac{T}{2},\;t\leq \frac{3T}{4})\;$***then***
$x_{i,j}\left(t+1\right)$ = $Best_{j}\left(t\right)\times P_{i,j}\left(t\right)\times randt$
***Else***
$x_{i,j}\left(t+1\right)$=$\;Best_{j}\left(t\right)-\eta_{i,j}\left(t\right)\times \varepsilon -R_{i,j}\left(t\right)\times randt$
***End if***
***End for***
***End for***
*t* = *t* + 1
***End while***

**Table A4:** Distance between mentioned cities of Mazandaran province (KM).

	Behshahr	Neka	Sari	Juybar	Qaemshahr	Pol Sefid	Savadkooh	Babolsar	Babol	Mahmood Abad
Behshahr	-	23	46	66	62	116	110	106	82	135
Neka	-	-	23	37	43	93	90	83	63	112
Sari	-	-	-	20	20	70	65	60	40	89
Juybar	-	-	-	-	20	70	65	34	43	75
Qaemshahr	-	-	-	-	-	50	45	40	20	69
Pol Sefid	-	-	-	-	-	-	10	90	70	119
Savadkooh	-	-	-	-	-	-	-	86	65	110
Babolsar	-	-	-	-	-	-	-	-	20	41
Babol	-	-	-	-	-	-	-	-	-	49
Mahmoudabad	-	-	-	-	-	-	-	-	-	-

**Table A5:** Tuning other parameters of model.

Parameters	Values	Units	Parameters	Values	Unit
capa_it_	Uniform (7000,8000)	Kilogram	*capf_f_*	Uniform (70,80)	Ton
chj_t_	Uniform (70,80)	Dollar per Ton	*pb_b_*	Uniform (300,350)	Kilogram
cpa_i_	Uniform (600,650)	Dollar per Ton	*cpj_j_*	Uniform (55,65)	Dollar per Ton
dct_p1ct_	Uniform (400,500)	Kilogram	*dct_p_*_2*ct*_	Uniform (30,35)	Ton
dco_p1ct_	Uniform (350,400)	Kilogram	*dco_p_*_2*ct*_	Uniform (25,30)	Ton
dk_kt_	Uniform (30,40)	Kilogram	*cpo_o_*	Uniform (200,300)	Dollar per Ton
du_ut_	Uniform (20,30)	Kilogram	*di_it_*	Uniform (200,300)	Kilogram
dm_mt_	Uniform (20,30)	Kilogram	*capj_j_*	Uniform (20,30)	Ton
pcap_pft_	Uniform (90,100)	Ton	*πs_s_*	Uniform (200,220)	Kilogram
*capr*_rt_	Uniform (20,100)	Megawatt hour	*πo_o_*	Uniform (200,220)	Kilogram
*ctax*_r_	Uniform (15,20)	Dollar per Megawatt hour	*vi_i_*	Uniform (15,20)	Person per ton
prm_pmt_	Uniform (2,5)	Dollar per Kg	*fcj_j_*_2_	Uniform (3,4)	Person
prf_pft_	Uniform (1.5,4.5)	Dollar per Kg	*fcs_s_*	Uniform (70,000,80,000)	Dollar
πb_b_	Uniform (200,220)	Kilogram	*fjo_o_*_2_	Uniform (5000,6000)	Dollar
cpo_ot_	Uniform (200,220)	Dollar per ton	*fcb_b_*	Uniform (500,000,600,000)	Dollar
po_o2_	Uniform (400,450)	Kilogram	*fjb_b_*	Uniform (4,5)	Person
ps_s_	Uniform (100,150)	Kilogram	*VJQ_pf_*	Uniform (10,12)	Person per ton

**Table A6:** The orthogonal array L9 and computational results for MOSA.

Run	Npop	Maxit	T0	Alpha	Response
1	1	1	1	1	2.49 × 10^−5^
2	1	2	2	2	2.62 × 10^−5^
3	1	3	3	3	2.58 × 10^−5^
4	2	1	2	3	2.53 × 10^−5^
5	2	2	3	1	1.74 × 10^−5^
6	2	3	1	2	1.68 × 10^−5^
7	3	1	3	2	1.21 × 10^−5^
8	3	2	1	3	1.45 × 10^−5^
9	3	3	2	1	1.32 × 10^−5^

**Table A7:** The orthogonal array L9 and computational results for MOPSO.

Run	Npop	Maxit	Phi1	Phi2	Response
1	1	1	1	1	1.54 × 10^−5^
2	1	2	2	2	1.36 × 10^−5^
3	1	3	3	3	1.33 × 10^−5^
4	2	1	2	3	1.73 × 10^−5^
5	2	2	3	1	1.28 × 10^−5^
6	2	3	1	2	1.09 × 10^−5^
7	3	1	3	2	1.03 × 10^−5^
8	3	2	1	3	1.3 × 10^−5^
9	3	3	2	1	1.22 × 10^−5^

**Table A8:** The orthogonal array L9 and computational results for MOGWO.

Run	Npop	Maxit	IR	Response
1	1	1	1	1.75 × 10^−5^
2	1	2	2	2.11 × 10^−5^
3	1	3	3	3.06 × 10^−5^
4	2	1	2	2.94 × 10^−5^
5	2	2	3	1.77 × 10^−5^
6	2	3	1	1.88 × 10^−5^
7	3	1	3	1.19 × 10^−5^
8	3	2	1	1.32 × 10^−5^
9	3	3	2	1.63 × 10^−5^

**Table A9:** The orthogonal array L9 and computational results for MORSO.

Run	Npop	Maxit	Alpha	Beta	Response
1	1	1	1	1	2.92 × 10^−5^
2	1	2	2	2	2.57 × 10^−5^
3	1	3	3	3	1.67 × 10^−5^
4	2	1	2	3	1.59 × 10^−5^
5	2	2	3	1	1.63 × 10^−5^
6	2	3	1	2	1.27 × 10^−5^
7	3	1	3	2	1.21 × 10^−5^
8	3	2	1	3	1.09 × 10^−5^
9	3	3	2	1	1.12 × 10^−5^
